# An integrated framework for antimicrobial resistance: links with climate change and vulnerability

**DOI:** 10.3389/fpubh.2025.1679189

**Published:** 2026-02-03

**Authors:** Estibaliz Baroja, Inmaculada Batalla, Maria Jose Sanz, Aline Chiabai

**Affiliations:** 1 Basque Centre for Climate Change (BC3), Leioa, Spain; 2 Plentzia Marine Station, University of the Basque Country (PiE-UPV/EHU), Plentzia, Spain; 3 Ikerbasque, Basque Foundation for Science, Bilbo, Spain

**Keywords:** AMR, climate change, IPCC risk framework, mDPSEEA, vulnerability

## Abstract

Antimicrobial resistance (AMR) has been extensively studied in clinical settings; however, research on the environmental aspects of AMR is relatively new. Recently, there has been growing interest in the relationship between climate change and AMR, yet evidence linking AMR to climate change and potential environmental transmission is very limited. Even less is understood about how vulnerabilities may exacerbate exposure and associated health risks. This study aims to compile literature on recent research on how climate change exacerbates risks associated with AMR. The study builds a framework based on this review that connects the amplifying effects of climate change to AMR risk using the modified DPSEEA (mDPSEEA) model. Additionally, the framework complements the mDPSEEA context by incorporating the *vulnerability* concept of the Intergovernmental Panel on Climate Change (IPCC) risk framework, which encompasses *susceptibility* and limited *coping capacity* to face exposure and potential health impacts of AMR. The integrated framework facilitates systemic analysis of the combined risk of climate change and AMR in its early stages, particularly within the *driver-pressure-state* interface. It also helps to identify vulnerable groups most likely to experience severe effects from AMR, such as the older adult(s), children, individuals with pre-existing chronic conditions, those at higher occupational risk of being colonised by antibiotic-resistant bacteria (ARB), and populations living in highly contaminated environments. The framework analysis emphasises that addressing AMR requires more than just isolated interventions; it demands a fundamental rethinking of public health planning and agendas. There is a need to develop strategies that coordinate various policy frameworks, including those about infectious diseases, chronic diseases and environmental hazards. Tackling climate change, pollution, and social inequalities is essential for combating AMR, as their interconnectedness cannot be overlooked.

## 1 Introduction

Antimicrobial resistance (AMR) is a natural mechanism by which microorganisms develop defence mechanisms against antimicrobial compounds. Today, AMR has become a global health problem as it threatens the effective treatment of infections with currently available drugs (1). According to the latest estimates of the global burden of AMR, bacterial AMR was associated with 4.71 million deaths worldwide in 2021, of which 1.14 million deaths were directly caused by AMR (2). The well-recognised study by O’Neill (145) estimated that, in the absence of specific actions, by 2050, 10 million people would die each year globally as a result of AMR, surpassing the number of deaths from cancer.

While the misuse and overuse of antibiotics, poor sanitation and infection control are widely recognised as a major cause of the increase in antibiotic resistance prevalence (3, 4), emerging research also highlights the role of other factors that contribute directly or indirectly to the persistence of AMR, from biological to ecological and social aspects, underlining its complexity as a multifactorial challenge (5, 6).

After consumption, up to 70–80% of antibiotics (per dose) are excreted in the urine and faeces (7). In human settings, these residues are frequently transported to wastewater treatment plants (WWTPs). As WWTPs are not capable of completely removing all antibiotics, these components are released into the effluents of these installations and enter the environment, where they can interact with environmental bacteria and promote selection pressures for antibiotic resistance (8).

Research on AMR has traditionally focused on clinical settings (9–12). However, in the last decade, other areas have gained considerable attention, for instance in recent years a growing body of research has focused on understanding the environmental dimension of the emergence and spread of AMR (13–19).

Aligned with these emerging research areas, recent efforts have been directed toward understanding the connections between climate change and AMR (20–25). Both issues, climate change and AMR, share critical characteristics such as “urgency, severity and global dimension” (26). Increasingly, it is recognised that these two challenges are not only related but deeply intertwined (26, 27). Indeed, it has been suggested that climate change should be one of the variables to be considered when constructing future scenarios to assess antibiotic resistance (28). Although the intersection presents significant yet poorly defined risks to global health security, international agencies are not prioritising research in this area (29).

Knowledge serves as a foundational step in the process of designing evidence-based interventions (30–32). Without robust knowledge production, the development of effective solutions becomes unattainable. Consequently, how research is supported is critical, as it directly influences the types of interventions that emerge.

In line with the research focus, interventions targeting AMR predominantly emphasise limiting and optimising antibiotic use (33–35). However, insufficient understanding of the interplay between AMR and other critical factors, such as climate change, can result in a limited range of interventions (36). For guiding the design of system perspective interventions, this study provides a scoping review with a conceptual framework for integrating AMR and climate change connections, as well as social and health inequities.

The framework employs the mDPSEEA model to frame AMR risk in the context of climate change, in particular, understanding how climate change promotes and disseminates AMR in the environment. The mDPSEEA model has been a widely recognised tool in the public health field and has also been proposed for tackling AMR (18, 37). Indeed, the DPSEEA model is catalogued as one of the most suitable frameworks for developing environmental health indicators for climate change and health (38). While addressing the links between climate change and AMR at early stages of the mDPSEEA, the proposed framework also introduces contextual factors amplifying the risk of exposure and AMR-related health impacts. In this line, to better address the social dimensions of AMR, the framework builds on the vulnerability concept outlined in the latest IPCC risk framework (39–41). This complementation provides a more nuanced understanding of how the social dimension shapes the risk of AMR to guide the design of targeted interventions.

## 2 Methodology

This study comprises two main components: a literature review and a conceptual framework. This section outlines the literature review process and includes two subsections detailing the nature of the models (mDPSEEA and IPCC risk framework) applied in the design of the proposed framework.

### 2.1 Literature review

A hybrid (scoping-critical) literature review has been carried out to build the integrated conceptual framework. Scoping reviews are useful to map evidence in a field with heterogeneous findings. They are usually used to map existing knowledge, gaps, clarify key concepts, characterise key elements related to a concept, suggest future directions in the research area and as precursors of systematic reviews (42, 43). In this process, research questions are typically used to guide the literature search (42–44). On the other side, critical or narrative review studies aim to understand the subjects, concepts, and perspectives on a topic of interest, but without following specific strategies or protocols (45, 46). Critical reviews are commonly used to gain a broad understanding of a topic in emerging fields, to build conceptual frameworks, or to synthesise information to inform policymakers (47–51).

Web of Science and Google Scholar were used to search for the studies. Diverse Boolean keyword search strategies were used for compiling studies fitting the different stages of the integrated model. For example, in the stage of Hazard (*driver-pressure-state*) (“climate change” OR “heavy precipitation” OR “storm” OR “temperature” OR “flooding”) AND (“AMR” OR “ARG” OR “infectious disease” OR “infection” OR “colonisation”). The selection criteria for the review were peer-reviewed articles; no strict temporal limits were imposed, but emphasis was placed on recent work (last ~10 years) to capture the current state of the field. Only studies published in English and available in full text were considered for inclusion. While the review did not strictly adhere to PRISMA guidelines for systematic or scoping reviews, it applied several core principles of these methodologies—such as structured keyword searching, source tracking, and the exclusion of papers with limited relevance. The screening process was carried out in two stages: initial review of titles and abstracts, followed by full-text assessment to determine each study’s relevance for informing the integrated conceptual framework.

68 studies were reviewed, and the characteristics of each study, the contribution to each of the framework stages and the supporting evidence were extracted into Supplementary Table A.

### 2.2 Description of reference models

#### 2.2.1 The mDPSEEA framework

The DPSEEA model was developed in 1995 by the World Health Organisation (WHO) as a methodological framework designed to support the development and assessment of policies related to the environment and health at various levels—local, regional, national, and international. Its primary strength lies in its capacity to summarise environmental health indicators into a simple, useful tool (37, 52, 53).

**Figure 1:**
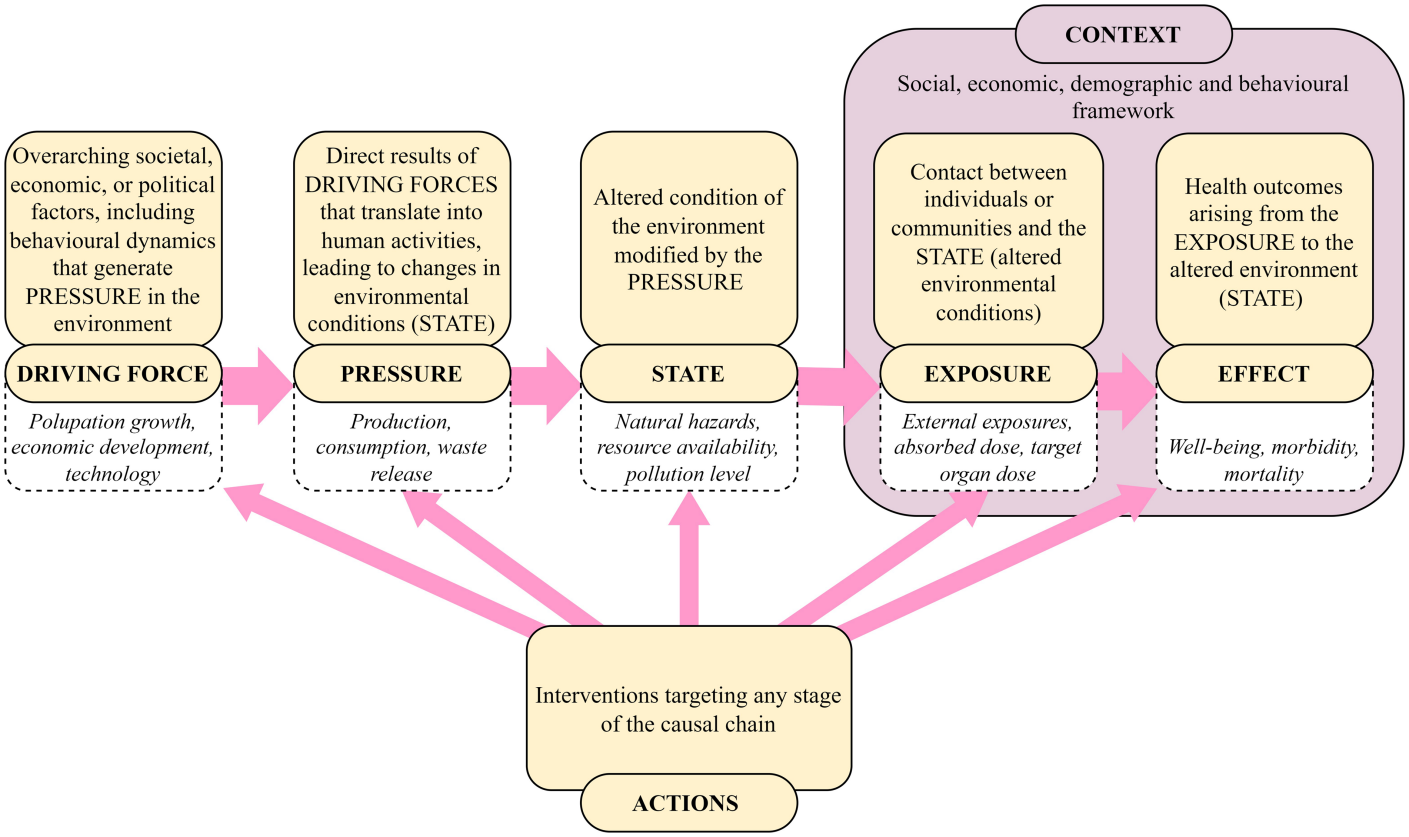
Illustration of the mDPSEEA model, description of the elements and examples. Adapted from Corvalán et al. (53) and Morris et al. (37).

The DPSEEA delineates a series of interconnected elements within a causal chain, defined as follows: *Driving Force-Pressure-State-Exposure-Effect-Action* (37, 52, 53) (Figure 1).

The model has successfully been applied to health risks associated with environmental pollution and climate change (54), and it has been previously applied to study the impacts of changes in the water environment on water-related diseases (55), and has also been proposed for AMR (17).

To incorporate factors capable of adapting to the particularities of the spatial dimension (local, regional or national), and in particular to incorporate the variability between exposure and health effects at the population level, Morris et al. (37) proposed an adapted version of the model, the modified DPSEEA or mDPSEEA (Figure 1). This version included the “context” as a set of external factors in the causal chain that influence whether pressures on the environment ultimately translate into positive or negative health outcomes for specific populations or sectors (37).

For the case of infectious diseases and AMR, we propose to frame the “context” through the lens of the vulnerability of the IPCC risk framework (AR5 and AR6).

#### 2.2.2 The IPCC risk framework: vulnerability

**Figure 2:**
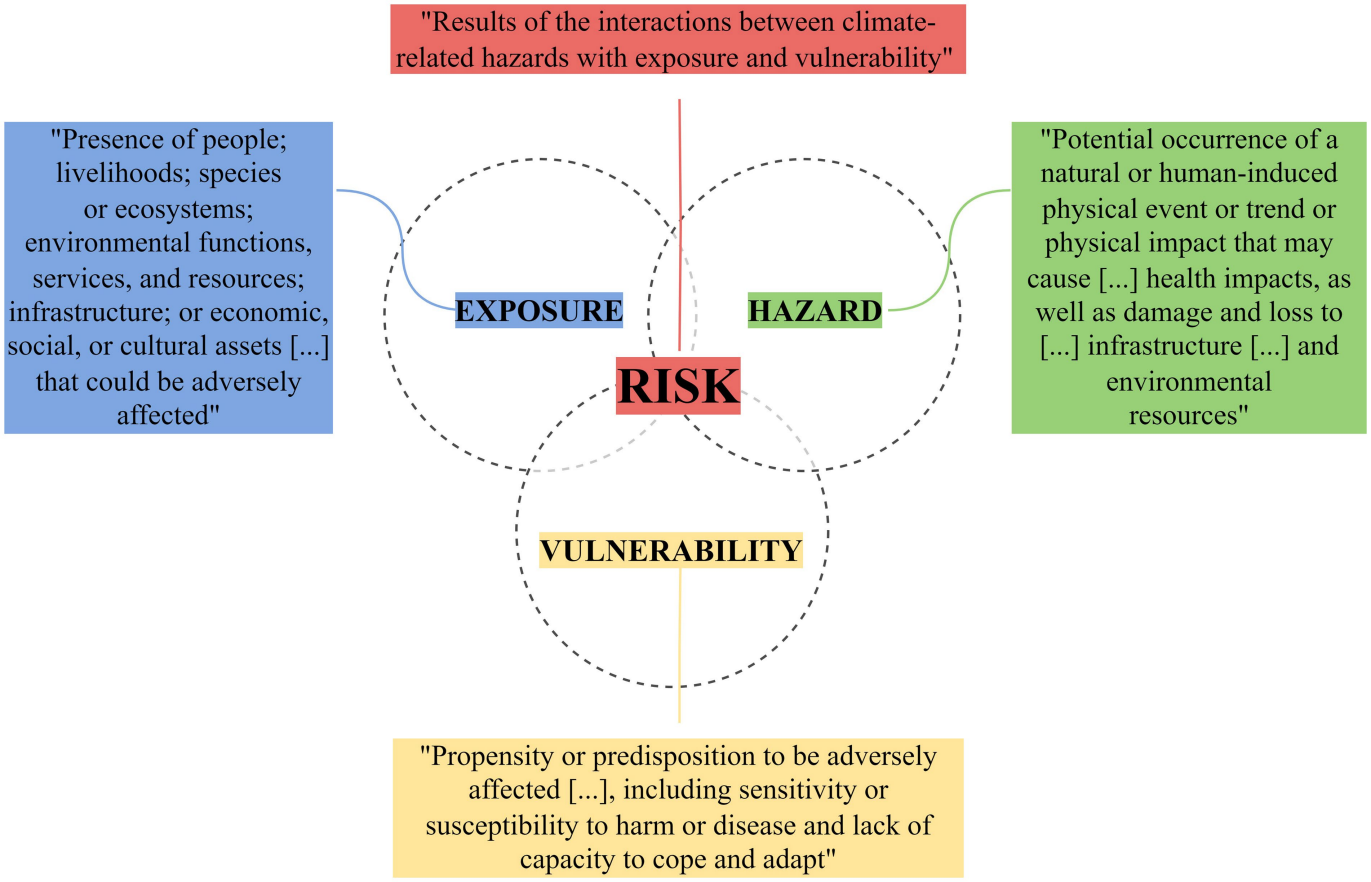
The IPCC climate-related risk framework (AR5 and AR6). Adapted from IPCC (40).

The concepts of risk and risk management have become increasingly central to climate change literature, research, practice and decision-making. In the new AR5 and AR6 reports, the IPCC provides a framework to better address how patterns of risks are shifting or accelerating due to climate change, this framework defines the risks derived from climate change as the results of the interactions between climate change related hazard with the exposure and the characteristics of the exposed population (39–41) (Figure 2).

By considering vulnerability as a contextual factor, this framework can elucidate the non-typical and potentially underestimated determinants of AMR risks. This integration provides a foundation for targeted interventions and research efforts by helping to identify population groups potentially more sensitive to AMR and factors that might influence the capacity to address the AMR.

## 3 Results

**Figure 3:**
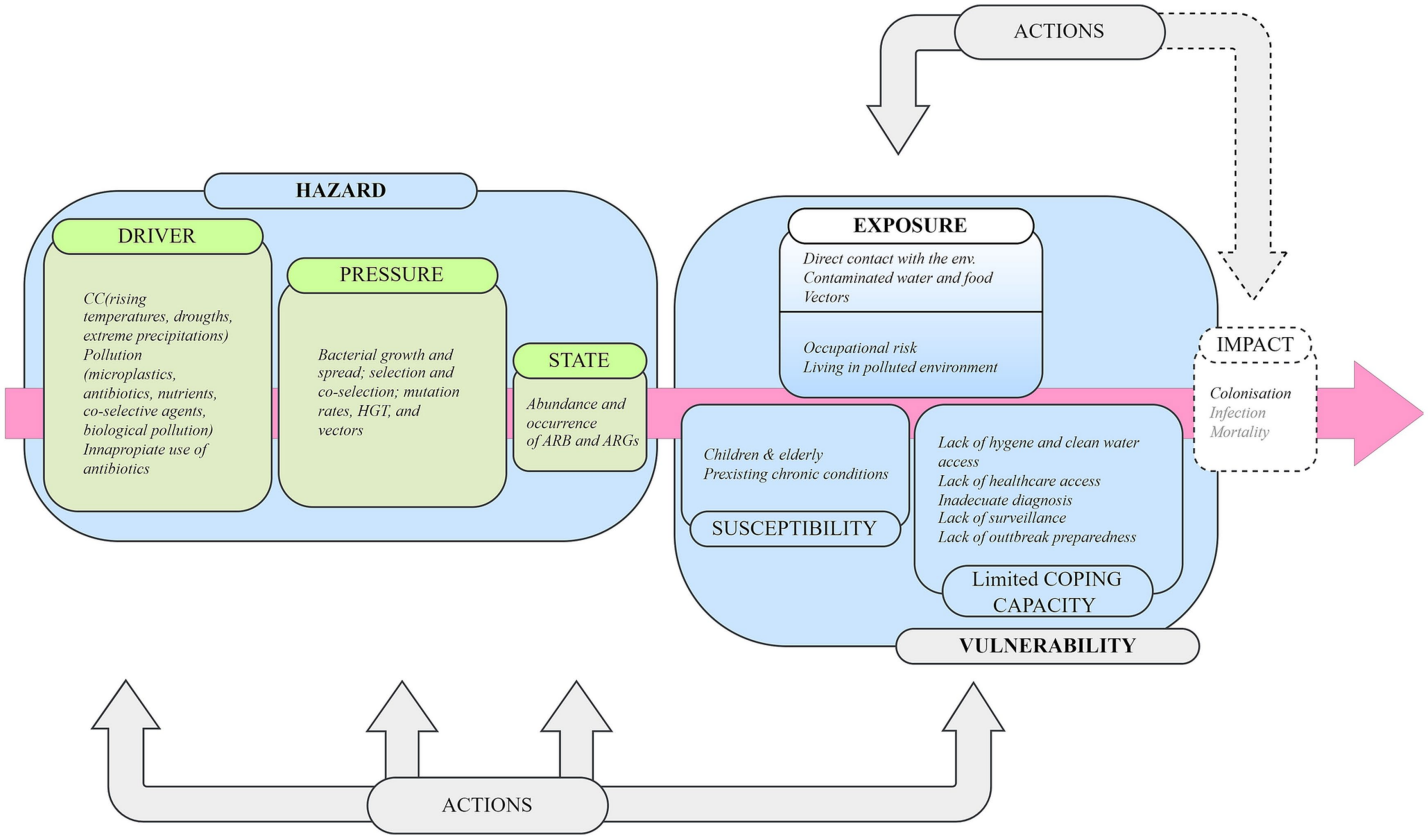
The integrated climate change-AMR framework is a conceptual model that helps to identify potentially vulnerable groups at risk from AMR.

This section develops the framework (Figure 3) through a narrative thread, structuring the reviewed literature according to the key elements of the proposed framework: the hazard (driver–pressure–state) and vulnerability in exposure–effect, while summarising the most relevant scientific evidence. The complete result of the literature review is compiled in Supplementary Table A.

Finally, to clarify, while AMR encompasses resistance mechanisms that various microorganisms develop against antimicrobial agents, the extensive body of literature predominantly addresses bacterial resistance specifically to antibiotics. Consequently, this study primarily compiles literature references on antibiotic resistance and uses the two terms interchangeably.

### 3.1 The hazard (driver–pressure–state)

Climate change is becoming recognised as a significant factor contributing to the emergence of AMR. Although research exploring the relationship between climate change and AMR is still at a preliminary stage, emerging studies suggest both phenomena are deeply and intimately interlinked (27). Recent efforts to further explore these linkages have been made by other authors as well (24, 56).

In this section, the Hazard is understood as how climate-related processes can intensify the selection and dissemination of AMR in the environment. To unpack these interlinkages, the main components of the links are organised according to three stages of the mDPSEEA framework: driver, pressure, and state.

- “Drivers” refer to the global-scale processes that influence the risk of AMR. In the context of climate change, we could identify rising temperatures, floods and droughts. In parallel, we have contaminating lifestyles and inappropriate use of antibiotics. These processes lead to the release of contaminants into the environment, including nutrients, microplastics, antimicrobials, antibiotics, and co-selective agents (such as heavy metals).
- “Pressures” refers to the mechanisms that these driving forces accelerate the selection and spread of AMR. Key examples include bacterial growth and spread, co-selection and selection of resistant strains, increased mutation rates, horizontal gene transfer (HGT), and the proliferation of AMR vectors.
- “State” describes the outcome of these pressures, expressed as the distribution and prevalence of antibiotic-resistant bacteria (ARB) or antibiotic resistance genes (ARGs) in the environment.

In discussing how climate change contributes to the emergence of AMR, rising temperatures are a key factor. Environmental temperature affects bacterial survival and proliferation. Higher temperatures have been observed to favour the growth of bacteria like *Salmonella* and *Vibrio* species in the environment (57, 58).

At the microbiological scale, several mechanisms explain how temperature influences AMR development. Microorganisms have evolved sophisticated physiological mechanisms to adapt to elevated thermal conditions, which represent a stress factor damaging cellular functioning, one of which is the heat shock response (59). This response is activated to fight physiological damage caused by high temperatures, such as DNA damage, protein misfolding, and destabilisation of the outer membrane (60). Furthermore, resistance to heat stress can confer cross-protection against certain antibiotics and *vice versa,* as both stressors produce similar cellular damage, triggering overlapping physiological protection (23, 61). For instance, Rodriguez-Verdugo, Gaut and Tenaillon (61) found that *Escherichia coli* developed and fixed parallel mutations within the rpoB gene, which conferred different levels of rifampicin resistance, without being exposed to antibiotics, but after being exposed to 42.2 °C for 2000 generations at high temperature.

Elevated temperatures may also impact bacterial evolution by increasing mutation rates under specific conditions. A recent study on *E. coli* demonstrated that raising the temperature from 37 °C to 40 °C significantly increased mutation rates, leading to resistance against antibiotics such as ciprofloxacin and rifampicin. However, this effect was antibiotic-specific, so the studies highlighted the complexity of these findings (62). Another process potentially influenced by warmer temperatures is HGT, a critical mechanism through which bacteria exchange genetic material, ARGs. HGT is considered one of the primary drivers of AMR dissemination. A recent study (63) examined the effect of local temperature on plasmid transfer encoding antibiotic resistance, finding that high temperatures of approximately 41–45 °C significantly promote cell-to-cell plasmid transformation in *E. coli* (64). However, a recent study found that the effectiveness of gatifloxacin against *E. coli* significantly diminishes at 42 °C, while resistance increases 256-fold at 27 °C (65). These findings underscore the potential role of temperature in shaping the dynamics of bacterial evolution and resistance; however, the underlying mechanisms are at an early research stage.

At a larger ecological scale, studies have observed a long-term association between temperature increase and antibiotic resistance [EU: (22, 66, 67); US: (21); China: (68)]. Kaba et al. (66), in a cross-sectional study across 30 European countries, reported that a 0.5 °C increase in annual temperature change was associated with a 1.02-fold increase in the prevalence of carbapenem-resistant *Pseudomonas aeruginosa* (CRPA). Pärnänen et al. (67) found that ARG burden after wastewater treatment was significantly higher in the southern countries than in the northern countries in Europe, suggesting that southern countries may have a higher impact on the receiving environment, including warmer temperatures. McGough et al. (22) found that in European countries with ambient minimum temperatures 10 °C higher than others experienced faster increases in antibiotic resistance (from 0.33 to 1.2%) for *E. coli* and *K. pneumoniae* across all antibiotic classes. In the United States (US), MacFadden et al. (21), for every 10 °C increase in minimum temperature, a significant increase in resistant strains of 4.2, 2.2 and 2.7% has been observed for three relevant pathogenic species (*E. coli, Klebsiella pneumoniae* and *Staphylococcus aureus*). Li et al. (68) found that 1 °C increase in average ambient temperature was associated with a 1.14-fold increase in carbapenem-resistant *K. pneumoniae* (CRKP) prevalence and a 1.06-fold increase in CRPA prevalence. Interestingly, the authors found that the cumulative impact of year-to-year variations in ambient temperature had the most significant effect on antibiotic resistance when analysed over 4 years.

In addition to studies examining the direct effects of temperature on AMR, there are studies examining indirect pathways by which climate change could be promoting the emergence of AMR. Experts warn that the spread of AMR is more severe in extreme weather conditions, which are expected to increase due to climate change (27). Indeed, heavy rainfall can cause sewage systems to overflow, resulting in the direct release of untreated agricultural and human wastewater into the environment. This untreated water contains ARB, ARGs (69–74) and co-selective agents (75)—compounds such as metals and biocides, which are not antibiotics but can promote antibiotic resistance selective pressure (76, 77). For example, Carney et al. (71) observed that the abundance of ARGs in coastal waters can increase by up to 100-fold following sewer overflow events associated with wet weather. Similarly, Ahmed et al. (69) reported a 42% increase in the detection frequency of the integron genes IntI2 and IntI3, which play a central role in the dissemination of ARGs, in samples from storm drain outfalls during wet weather. In the same study, carbapenem-resistance genes and the beta-lactam resistance gene were detected exclusively under wet weather conditions. The results for heavy metals appear inconclusive, since their behaviour varies by element. Drozdova et al. (75) found that during wet weather periods, concentrations of As, Cr, Cd, Pb, Mn, and Fe increased in water samples. By contrast, concentrations of Zn, Cu, and Ni decreased under the same conditions, while Hg levels did not change significantly. In addition, wastewater has been identified as a potential source of microplastics (MPs) in various environmental compartments (78, 79). Recent studies found that MPs not only could promote the dissemination of ARB into the environment (80) but that exposure to MPs promotes the conjugation rates of four clinically relevant AMR plasmids by up to 200-fold (81). Finally, agricultural runoff can be a major source of nitrogen and phosphorus entering the environment (82), which do not directly promote AMR but could contribute to the emergence of AMR by creating favourable conditions for bacteria to thrive (83).

Another potential pathway yet to be explored by which climate change could disseminate AMR is the contribution of the infectious disease vectors. Many studies have examined how climate change-induced variations in temperature and precipitation influence vectors such as mosquitoes, ticks, and flies, which are critical in the transmission of infectious diseases like Zika, malaria, dengue fever, and Lyme disease (84–87). During droughts, there is an increased success rate of vectors, e.g., mosquitoes have fewer predators in drought conditions and breed more in sewage pits when there is a shortage of water (88). Similarly, for ARB, certain vectors such as flies, cockroaches and ticks have been identified as carriers of ARB (89, 90). Indeed, a recent whole-genome sequencing study proved arthropod-mediated transmission of multidrug-resistant Enterobacterales (MDRE) in hospitals in low- and middle-income countries (LMICs) (91).

In addition to specific pathways to AMR, there is a vast literature on the effects of climate change on infectious disease dynamics and risks. Although this literature does not focus exclusively on resistant infections, it is particularly relevant to this review, as the overall incidence of infections is expected to increase as a result of climate change. Reviews and meta-analyses have captured the numerous pathways through which, indirectly or directly, climate change may increase the risk of infectious diseases (92, 93); indeed, it has recently been estimated that climate hazards have exacerbated 58% of infectious diseases worldwide, impacting human health (94). For instance, the distance from the equator and socioeconomic factors have been associated with the risk of gram-negative bacteraemia in a sample of 22 cities (95). Indeed, it was found that humidity, monthly rainfall and temperature correlated with gram-negative bloodstream infections (96). Heavy precipitation and sea level rise, for example, increase the likelihood of flooding, which is closely linked to the spread of waterborne infections (94, 97). Indeed, flooding and drought are linked to phenomena such as displacements and overcrowding of people, which are risk factors for the spread of infectious diseases. Phenomena such as floods facilitate the transmission of infectious pathogens, including ARB (97–99). During droughts, water resources have to be shared among more people, and sanitary conditions are poorer. Overcrowding, poor hygiene and water scarcity are perfect breeding grounds for outbreaks of waterborne diseases (97–101).

Finally, population groups most vulnerable to climate change might face a higher risk of infections. For example, people with pre-existing chronic conditions, already vulnerable to heatwave risks or infectious diseases (e.g., tuberculosis, salmonellosis, malaria) (27), might be at higher risk of further infections (94). Climate change is also putting pressure on sanitation systems and health care and is exacerbating social and health inequalities, which further worsen the risk of infections (102, 103). These examples illustrate some of the vulnerabilities linked to AMR within the context of climate change. The subsequent section will expand on this by addressing additional AMR vulnerabilities.

So far, we have discussed the implications of climate change as a cause of the increase and spread of antibiotic resistance and infectious diseases. However, the relationship between climate change and antibiotic resistance is extremely complex and might not be unidirectional. For example, the administration of antibiotics to cattle or the presence of antibiotics in river systems promotes methane production in methane-producing microorganisms (104, 105).

### 3.2 Vulnerability in exposure-effect

Although the available literature is scarce, this section aims to provide an overview of the potential exposure to environmental AMR and help to identify potentially vulnerable groups when facing AMR health risk. To do so, incorporate the last two elements of the mDPSEEA, *exposure–effect,* the concept of *vulnerability* from the IPCC risk framework, to better contextualise the groups at highest risk.

- “Exposure” refers to how humans get exposed to ARB and ARGs from the environment. They include direct contact with environmental AMR through recreational activities, consuming contaminated food and drinking water and contact with potential vectors of ARB and ARG.
- “Effect” includes colonisation and infections—such as gastrointestinal, respiratory tract, soft tissue, and wound infections—as well as mortality attributable to or associated with AMR-resistant pathogens.
- “Vulnerability” is the propensity of being adversely affected by AMR.

While the *exposure–effect* stage has been extensively studied in clinical settings, the link between environmental exposure to AMR and human health outcomes remains poorly understood. Recent reviews characterised the current evidence on the risks of environmental exposure to AMR (17, 18, 106). Stanton et al. (18) identified the main exposure pathways studied as the consumption or ingestion of raw food and water contaminated with ARB or ARGs. The most frequently investigated health outcomes were infection and overall exposure risk, and the principal type of studies was risk assessments. The scarce literature on the direct transmission of AMR from the environment to humans highlights the complexity in conducting observational studies in this area; however, the literature indicates a potential risk of exposure.

In the proposed framework, the health outcome depends on the intensity and frequency of *exposure* (123) and the *vulnerability* of the exposed population, which encompasses variances in exposure to hazards, sensitivity or *susceptibility* and *coping capacity*. This framework contextualises these components for AMR risks.

Not all individuals have the same probability of being exposed to environmental AMR. One of the groups identified as being at higher risk of exposure is heavy recreational water users, such as surfers (107, 108). Leonard et al. (107) estimated water ingestion volumes during different water sports and reported that, in England and Wales, one in every 6.3 million water sport sessions resulted in the ingestion of at least one strain of *E. coli* resistant to third-generation cephalosporins, a clinically important class of antibiotics. In a subsequent study, Leonard et al. (108) found that surfers were at higher risk of colonisation with *E. coli* carrying the bla_CTX-M_ gene compared with non-surfers (6.3 and 1.5%, respectively). Nevertheless, as this field of research is still in its early stages, there are no conclusive findings; for instance, a more recent study reported lower colonisation rates or ARB among regular swimmers (109).

Consumption of raw foods directly exposed to environmental sources represents a potential route of human exposure to AMR. In particular, filter-feeding shellfish, such as oysters, are of concern, as studies have detected both ARB and ARG in these products (110, 111). In addition, Svanenik et al. (111), who conducted whole-year, coast-wide surveillance of AMR in *E. coli*, identified extended-spectrum cephalosporin (ESC)-resistant strains, associated with human and animal infections, in bivalve molluscs suitable for market sale, including scallops and flat oysters. Through the use of manure as a fertiliser, among other sources, AMR is spread into soil and crops (8, 112). In this context, the consumption of raw vegetables has been identified as a potential exposure risk. For instance, a recent study of fresh vegetables from Swiss retailers found that 95% of the fresh vegetable samples contained ARGs (113).

Another potential higher risk group are those at occupational higher risk of acquiring ARB or ARGs from the environment. Landfills and WWTPs are widely recognised as key hotspots for the emergence of AMR (114, 115). The exposure of workers and nearby communities to ARB and ARGs via bioaerosols from these facilities is of particular concern (116, 117). Nevertheless, a recent study reported conflicting findings regarding the occupational risk of AMR, showing no increased ARG abundance in WWTP workers and identifying factors such as country of residence and recent antibiotic use as the strongest determinants of ARG abundance (118). Another potential occupational exposure is that experienced by farm workers, who are at higher risk of encountering AMR (119), for example, in chicken farms, high concentrations of bacterial aerosols were found (3.117 × 10^4^ CFU/m^3^), exceeding the corresponding limit (120).

Finally, another group at high exposure risk are people living in heavily polluted environments, e.g., polluted rivers, who do not have access to clean drinking water and where hygiene measures are limited (121). An ecological study analysing 1,589 metagenomes from 26 countries found a higher prevalence of ARGs in low- and middle-income countries (LMICs), and that greater access to improved water and sanitation was associated with lower ARG abundance (effect estimate −0·22, [95% CI –0·39 to −0·05]). A clearer understanding of potential exposure pathways is required, as recent studies have reported the presence of ARGs and ARB in conventionally treated drinking water, even under low-level chlorination, highlighting the need for further investigation into treatment efficacy and residual risks (122).

The *susceptibility* of the individuals and the population is key in determining the risk of *exposure* and severity of infections, or health outcomes (123). One significant factor determining health outcomes from ARB infections is demographics, particularly age (2, 124, 125). Naghavi et al. (2), in a study covering 204 countries and territories between 1990 and 2021, estimated that AMR-related deaths declined by more than 50% among children under five, but increased by over 80% in adults aged 70 years and older.

Nevertheless, it has been acknowledged that the prevalence of ARB infections is high in children. In 2022, an estimated 3 million children worldwide died from AMR-related infections. This burden was particularly pronounced in low- and middle-income countries (LMICs), where resistance rates were highest and data collection remained limited (125, 126); this may be because their immune systems are not yet fully developed. Similarly, older adult(s) people are also more vulnerable due to immunosenescence and multimorbidity, which might translate into more severe consequences of ARB infections (2, 127, 128). In their review, Theodorokis et al. (25) identified underlying factors for AMR-related health in the older adult(s): immunosenescence, polypharmacy, sarcopenia and malnutrition, frailty and decreased mobility, cognitive impairment, frequent hospitalisations and long-term care facilities (LTCF) residency, and multimorbidity. Indeed, some chronic pathologies can increase the risk of certain infections. For instance, individuals with chronic respiratory problems, such as asthma, are potentially more vulnerable to infectious respiratory diseases (129). Similarly, people with diabetes have been found to be at a higher risk of infections (130, 131).

Thirdly, *coping capacity* refers to the ability of communities or institutions to face the “exposure” and “impacts.” Limited *coping capacity* at the community level, socio-economic factors such as low income and living in remote rural areas, might affect access to the health care system and proper treatment for ARB infections, which might result in a higher risk of developing severe symptoms after infection (132–135). For instance, Cooper et al. found significant spatial autocorrelation between the area deprivation index (ADI) and the prevalence of AMR organisms in clinical patients. At a more institutional scale, other barriers may reduce the capacity to adapt to AMR risks, for instance, lack of adequate diagnostics, lack of surveillance or lack of preparedness for outbreaks (136).

These studies, though still scarce in the scientific literature, show the importance of vulnerability factors linked to determinants of human health beyond the healthcare system, and beyond purely biomedical concepts.

## 4 Discussion

The integrated framework offers a theoretical approach to synthesising knowledge on the complex interactions between antimicrobial resistance (AMR) and climate change at early stages of the mDPSEEA framework, as well as between AMR and vulnerability in the exposure–effect stages. While this framework provides valuable insights into the interconnections between these phenomena, several limitations must be acknowledged to contextualise the results. A key limitation is that AMR research has traditionally focused on clinical settings, resulting in a limited evidence base linking AMR to climate change, the potential transmission of environmental AMR to humans, and the influence of social and health inequalities on outcomes.

In the framework, climate change is acting as a *driver* of resistance through several pathways and scales. For instance, temperature increases have been acknowledged as a risk factor promoting AMR. At microbiological levels, warmer temperatures can promote bacterial growth, mutation rates, and the exchange of ARGs (57, 58, 62–64). In addition, experts claim that some of the mechanisms that micro-organisms have evolved to adapt to high temperatures (e.g., the heat shock response) may also provide cross-protection against certain antibiotics, which could translate into an increased ability to cope with certain antibiotics in warmer conditions (23, 61). At the ecological level, studies indicate that rising local temperatures are linked to the spread and distribution of antibiotic resistance (21, 22, 67).

In addition to direct connections between climate change and AMR, there are indirect pathways through which climate change contributes to the spread of AMR. For instance, changing climate conditions, including rising temperatures and shifting precipitation patterns, influence the epidemiology of infectious diseases. These changes can alter the spatial distribution of disease vectors, such as mosquitoes and ticks, which are responsible for transmitting illnesses like malaria (137). Furthermore, they can expand the geographic range of pathogens, as observed with *Vibrio vulnificus* (138), which might potentially increase opportunities for the spread of AMR. Another indirect pathway is that heavy rainfall resulting from climate change can cause sewage systems to overflow, leading to the release of untreated wastewater into the environment, which harbours ARBs, ARGs and co-selective agents that can promote AMR (74).

On the other hand, this framework also facilitates the identification of the heterogeneous of exposure and the effect of AMR. This is particularly helpful as it is able to target various aspects of social context, including demographics and pre-existing health conditions. To do so, the framework incorporates the IPCC risk framework (AR5 and AR6), which conceptualised climate-related risk as the result of three interacting components: hazard, exposure, and *vulnerability*.

In other words, the framework highlights the role of the social dimension in determining health outcomes. While historically, interventions on the health impacts of AMR have been approached from an exposure point of view, the framework presents another, less explored, but potentially important pathway through the concept of *vulnerability*. This is composed of two elements: limited *coping capacity*, which is the limitations to cope with exposure and impacts, it includes socio-economic level, hygene, access to the healthcare system and poor infection surveillance and control; and sensitivity or *susceptibility*, which refer to health disparities that may increase the risk of infection and developing severe disease following infection. These conditions include age and weakened general health status due to pre-existing conditions.

This latter global challenge represents an increasing epidemic of non-communicable diseases (NCDs), the largest cause of death in the world, and is placing a huge socioeconomic burden on society. The GBD 2019 study estimated that in 2019 almost 90% of deaths and more than 80% of DALYs in European regions were attributable to non-communicable diseases (NCDs) (e.g., cardiovascular, neoplasm, chronic respiratory, diabetes and kidney, and digestive) (139, 140). We have seen that the increasing risk of chronic diseases for larger proportions of the population represents a vulnerability factor for AMR threats. People with pre-existing chronic conditions have a compromised immune system and will be highly vulnerable to AMR risks of infection and death. Chronic patients are also more likely to have long stays in nursing homes or hospitals, where there are higher risks of acquiring resistant bacterial infections and a higher probability of being prescribed antibiotics (127, 141). WHO warns that we may enter a post-antibiotic era where bacterial infectious diseases that up till now were successfully treated with antibiotics could lead to severe illness and premature deaths (142). In sum, we are moving towards a scenario of an increase in both chronic and infectious diseases, which will be a deadly combination in terms of population resilience in the face of the many global health threats.

Additionally, although not extensively reviewed, the framework visualizes potential entry points for interventions along the mDPSEEA causal chain, thereby expanding the range of strategies available to address AMR. Indeed, the framework serves as a foundational tool to guide policymakers in identifying and coordinating targeted areas for intervention. One of the key findings provided by the framework is the complexity underlying AMR and how all the connections among the different stage and scales of the framework conform a complex system.

Recognising AMR as a complex system means recognising unexpected outcomes or unintended consequences. Paradoxes emerge because even a single pathway often encompasses subsystems that interact with higher-level drivers, often simultaneously. A great example of this is the heterogeneous evidence related to the risk of antibiotic-resistant bacteria ARB coming from recreational water exposure. While a study found that surfers face higher risks of colonisation by ARB in coastal waters (108), another reported lower colonisation rates among regular swimmers (109). The latter suggested that discrepancies may reflect differences in study populations, water quality, or analytical methods. Moreover, the authors hypothesised that another factor to consider could be that exposure to high-quality water might have protective effects by enhancing microbiome diversity, potentially reducing susceptibility to ARB in future low-quality water exposures. So, incorporating the human microbiome when exposed to environmental ARB opens a box of a full network of interactions, i.e., a subsystem.

Unintended consequences emerge because complex systems are characterised by feedback loops and unexpected synergies. This review examined how climate change may promote the selection and dissemination of AMR (Supplementary Table A). Yet, the reverse relationship might also be possible: antibiotics have been linked to increased greenhouse gas (GHG) emissions through their stimulation of methane production in freshwater microorganisms (104). Beyond antibiotics themselves, the production of antibiotics as part of the pharmaceutical industry contributes to greenhouse gas (GHG) emissions, further exemplifying systemic feedback loops. A recent study estimated that in the US, the unnecessary prescription of antibiotics (which is around 30–50% of total antibiotics prescribed) accounted for approximately 1,887,374 tons of CO₂ emissions in 2022 (143) These examples highlight the importance of adopting a systems perspective to anticipate and prevent unintended outcomes. For instance, climate adaptation strategies such as wastewater reuse for irrigation, although beneficial for water conservation, may inadvertently spread antibiotic residues, resistant bacteria, and resistance genes across environmental compartments (144).

In this uncertain context, and given the urgency of the issue, policy action cannot remain blocked while awaiting complete evidence base. Mitigation must be central, with particular emphasis on reducing hazards across the causal chain, whether drivers, pressures, or states. This includes preventing pollutants such as plastics, antibiotics, co-selective agents, and nutrients from entering the environment. At the same time, research must continue to unravel these complex interactions and inform targeted adaptive measures.

In sum, AMR represents one of the most pressing public health challenges of our time: a multifactorial and complex problem that cannot be addressed through single-target or sector-specific approaches. Effective responses demand systemic, structural action across multiple levels of the causal chain. This requires a fundamental reorientation of public health agendas, one that prioritises source control while fostering coordination across diverse policy domains, including health, environment, and social policy. Only through such integrated approaches can we simultaneously mitigate environmental threats (e.g., pollution, climate change) and reduce vulnerabilities in populations (e.g., health inequities, limited access to care).

## 5 Conclusion

This framework is a foundational step toward integrating interlinks between global environmental and health hazards, such as climate change and AMR. Even notable knowledge gaps remain in the field; this framework could serve as a knowledge platform for discussion and collaboration between different stakeholders to design plans that combine multiple actions to promote integrated AMR actions. These discussions can, in turn, promote inter- and transdisciplinary research opportunities between biomedical and non-biomedical fields on the interaction of climate change and AMR.

In addition, the framework provides a multi-hazard perspective by integrating other public health threats, such as chronic diseases or socioeconomic inequalities. The proposed conceptual framework is an open groundwork that will be nourished by new knowledge available in the future.

## References

[1] Menz BD Charani E Gordon DL Leather AJM Moonesinghe SR Phillips CJ. Surgical antibiotic prophylaxis in an era of antibiotic resistance: common resistant Bacteria and wider considerations for practice. Infect Drug Resist. (2021) 14:5235–52. doi: 10.2147/IDR.S319780, 34908856 PMC8665887

[2] Naghavi M Vollset SE Ikuta KS Swetschinski LR Gray AP Wool EE . Global burden of bacterial antimicrobial resistance 1990–2021: a systematic analysis with forecasts to 2050. Lancet. (2024) 404:1199–226. doi: 10.1016/S0140-6736(24)01867-1, 39299261 PMC11718157

[3] ECDC (2017) Factsheet for the general public - Antimicrobial resistance, Antimicrobial resistance. Available online at: https://www.ecdc.europa.eu/en/antimicrobial-resistance/facts/factsheets/general-public (Accessed: 7 November 2024).

[4] Mancuso G Midiri A Gerace E Biondo C. Bacterial antibiotic resistance: the Most critical pathogens. Pathogens. (2021) 10:1310. doi: 10.3390/pathogens10101310, 34684258 PMC8541462

[5] Nguyen MT Maeda T Mohd Yusoff MZ Ogawa HI. Effect of azithromycin on enhancement of methane production from waste activated sludge. J Ind Microbiol Biotechnol. (2014) 41:1051–9. doi: 10.1007/s10295-014-1446-z, 24793122

[6] Salam MA al-Amin MY Salam MT Pawar JS Akhter N Rabaan AA . Antimicrobial resistance: A growing serious threat for global public health. Healthcare. (2023) 11:1946. doi: 10.3390/healthcare11131946, 37444780 PMC10340576

[7] Baietto L Corcione S Pacini G Perri G D’Avolio A de Rosa F. A 30-years review on pharmacokinetics of antibiotics: is the right time for pharmacogenetics? Curr Drug Metab. (2014) 15:581–98. doi: 10.2174/1389200215666140605130935, 24909419 PMC4435065

[8] UNEP (2017) Frontiers 2017 emerging issues of environmental concern. DEW/2124/NA. Nairobi. Available online at: https://www.unep.org/resources/frontiers-2017-emerging-issues-environmental-concern (Accessed May 07, 2024).

[9] Cantón R Horcajada JP Oliver A Garbajosa PR Vila J. Inappropriate use of antibiotics in hospitals: the complex relationship between antibiotic use and antimicrobial resistance. Enferm Infecc Microbiol Clin. (2013) 31:3–11. doi: 10.1016/S0213-005X(13)70126-5, 24129283

[10] Dulon M Haamann F Peters C Schablon A Nienhaus A. MRSA prevalence in european healthcare settings: a review. BMC Infect Dis. (2011) 11:138. doi: 10.1186/1471-2334-11-138, 21599908 PMC3128047

[11] Magiorakos A-P Suetens C Monnet DL Gagliotti C Heuer OE EARS-Net Coordination Group and EARS-Net participants. The rise of carbapenem resistance in Europe: just the tip of the iceberg? Antimicrob Resist Infect Control. (2013) 2:6. doi: 10.1186/2047-2994-2-6, 23410479 PMC3691711

[12] Perez F Endimiani A Ray AJ Decker BK Wallace CJ Hujer KM . Carbapenem-resistant Acinetobacter baumannii and *Klebsiella pneumoniae* across a hospital system: impact of post-acute care facilities on dissemination. J Antimicrob Chemother. (2010) 65:1807–18. doi: 10.1093/jac/dkq191, 20513702 PMC2904665

[13] Allen HK Donato J Wang HH Cloud-Hansen KA Davies J Handelsman J. Call of the wild: antibiotic resistance genes in natural environments. Nat Rev Microbiol. (2010) 8:251–9. doi: 10.1038/nrmicro2312, 20190823

[14] Allen SE Boerlin P Janecko N Lumsden JS Barker IK Pearl DL . Antimicrobial resistance in generic *Escherichia coli* isolates from wild small mammals living in swine farm, residential, landfill, and natural environments in southern Ontario, Canada. Appl Environ Microbiol. (2011) 77:882–8. doi: 10.1128/AEM.01111-10, 21131524 PMC3028733

[15] EMA 2021 Reflection paper on antimicrobial resistance in the environment: Considerations for current and future risk assessment of veterinary medicinal products. Amsterdam, The Netherlands: European Medicines Agency.

[16] Larsson DGJ Andremont A Bengtsson-Palme J Brandt KK de Roda Husman AM Fagerstedt P . Critical knowledge gaps and research needs related to the environmental dimensions of antibiotic resistance. Environ Int. (2018) 117:132–8. doi: 10.1016/j.envint.2018.04.041, 29747082

[17] Stanton IC Bethel A Leonard AFC Gaze WH Garside R. What is the research evidence for antibiotic resistance exposure and transmission to humans from the environment? A systematic map protocol. Environ Evid. (2020) 9:12. doi: 10.1186/s13750-020-00197-6, 32518638 PMC7268584

[18] Stanton IC Bethel A Leonard AFC Gaze WH Garside R. Existing evidence on antibiotic resistance exposure and transmission to humans from the environment: a systematic map. Environ Evid. (2022) 11:8. doi: 10.1186/s13750-022-00262-2, 35308196 PMC8917330

[19] UNEP (2023) ‘Tackling antimicrobial resistance: Stopping pollution at source’, UNEP. Available online at: http://www.unep.org/news-and-stories/speech/tackling-antimicrobial-resistance-stopping-pollution-source (Accessed May 06, 2024).

[20] Allel K. Exploring the relationship between climate change and antimicrobial-resistant bacteria: to what extent does this present a current and long-term threat to population health? Int J Climate Change. (2021) 13:27. doi: 10.18848/1835-7156/CGP/v13i01/27-37

[21] MacFadden DR McGough SF Fisman D Santillana M Brownstein JS. Antibiotic resistance increases with local temperature. Nat Clim Chang. (2018) 8:510–4. doi: 10.1038/s41558-018-0161-6, 30369964 PMC6201249

[22] McGough SF MacFadden DR Hattab MW Mølbak K Santillana M. Rates of increase of antibiotic resistance and ambient temperature in Europe: a cross-national analysis of 28 countries between 2000 and 2016. Eurosurveillance. (2020) 25:1900414. doi: 10.2807/1560-7917.ES.2020.25.45.1900414, 33183408 PMC7667635

[23] Rodríguez-Verdugo A Lozano-Huntelman N Cruz-Loya M Savage V Yeh P. Compounding effects of climate warming and antibiotic resistance. iScience. (2020) 23:101024. doi: 10.1016/j.isci.2020.101024, 32299057 PMC7160571

[24] Magnano San Lio R Favara G Maugeri A Barchitta M Agodi A. How antimicrobial resistance is linked to climate change: an overview of two intertwined global challenges. Int J Environ Res Public Health. (2023) 20:1681. doi: 10.3390/ijerph20031681, 36767043 PMC9914631

[25] Bavel B. van van Bavel B Berrang-Ford L Moon K Gudda F Thornton AJ ., 2024 Intersections between climate change and antimicrobial resistance: a systematic scoping review Lancet Planet Health 8 e1118–e1128 doi: 10.1016/S2542-5196(24)00273-0 39674199

[26] Harring N Krockow EM. The social dilemmas of climate change and antibiotic resistance: an analytic comparison and discussion of policy implications. Humanit Soc Sci Commun. (2021) 8:125. doi: 10.1057/s41599-021-00800-2

[27] Burnham JP. Climate change and antibiotic resistance: a deadly combination. Therapeutic Adv Infect Disease. (2021) 8:204993612199137. doi: 10.1177/2049936121991374, 33643652 PMC7890742

[28] Grilo ML Pereira A Sousa-Santos C Robalo JI Oliveira M. Climatic alterations influence bacterial growth, biofilm production and antimicrobial resistance profiles in Aeromonas spp. Antibiotics. (2021) 10:1008. doi: 10.3390/antibiotics10081008, 34439058 PMC8389027

[29] Bertagnolio S Dobreva Z Centner CM Olaru ID Donà D Burzo S . WHO global research priorities for antimicrobial resistance in human health. Lancet Microbe. (2024) 5. doi: 10.1016/S2666-5247(24)00134-4, 39146948 PMC11543637

[30] Hosseini M-S Jahanshahlou F Akbarzadeh MA Zarei M Vaez-Gharamaleki Y. Formulating research questions for evidence-based studies. J Med Surg Public Health. (2024) 2:100046. doi: 10.1016/j.glmedi.2023.100046

[31] Schwenkenbecher A Hewitt CL Heesen R Campbell ML Fritsch O Knight AT . Epistemology of ignorance: the contribution of philosophy to the science-policy interface of marine biosecurity. Front Mar Sci. (2023) 10. doi: 10.3389/fmars.2023.1178949

[32] Titler MG. The evidence for evidence-based practice implementation In: Hughes RG, editor. Patient safety and quality: An evidence-based handbook for nurses. Rockville (MD): Agency for Healthcare Research and Quality (US) (2008) 21328752

[33] Ayorinde A Ghosh I Shaikh J Adetunji V Brown A Jordan M . Improving healthcare professionals’ interactions with patients to tackle antimicrobial resistance: a systematic review of interventions, barriers, and facilitators. Front Public Health. (2024) 12. doi: 10.3389/fpubh.2024.1359790, 38841670 PMC11150712

[34] Charani E McKee M Ahmad R Balasegaram M Bonaconsa C Merrett GB . Optimising antimicrobial use in humans – review of current evidence and an interdisciplinary consensus on key priorities for research. Lancet Regional Health - Europe. (2021) 7:100161. doi: 10.1016/j.lanepe.2021.100161, 34557847 PMC8454847

[35] Knowles R Chandler C O’Neill S Sharland M Mays N. A systematic review of national interventions and policies to optimize antibiotic use in healthcare settings in England. J Antimicrob Chemother. (2024) 79:1234–47. doi: 10.1093/jac/dkae061, 38507232 PMC11144483

[36] Nguyen-Thanh L Wernli D Målqvist M Graells T Jørgensen PS. Characterising proximal and distal drivers of antimicrobial resistance: an umbrella review. J Global Antimicrob Resist. (2024) 36:50–8. doi: 10.1016/j.jgar.2023.12.008, 38128730

[37] Morris GP Beck SA Hanlon P Robertson R. Getting strategic about the environment and health. Public Health. (2006) 120:889–903. doi: 10.1016/j.puhe.2006.05.022, 16965797

[38] Hambling T Weinstein P Slaney D. A review of frameworks for developing environmental health indicators for climate change and health. Int J Environ Res Public Health. (2011) 8:2854–75. doi: 10.3390/ijerph8072854, 21845162 PMC3155333

[39] IPCC. Climate change 2014: impacts, adaptation, and vulnerability: working group II contribution to the fifth assessment report of the intergovernmental panel on climate change. New York, NY: Cambridge University Press (2014).

[40] IPCC (2014) ‘Summary for policymakers’. In: Edenhofer OR, Pichs-Madruga Y, Sokona E, Farahani S, Kadner K, Seyboth, A. (eds.). Climate change 2014: mitigation of climate change. Contribution of working group III to the fifth assessment report of the intergovernmental panel on climate change. Cambridge, United Kingdom and New York, NY, USA: Cambridge University Press.

[41] IPCC (2022) ‘Climate change: a threat to human wellbeing and health of the planet. Taking action now can secure our future — IPCC’. Available online at: https://www.ipcc.ch/2022/02/28/pr-wgii-ar6/ (Accessed: 30 October 2023).

[42] Peters MDJ Marnie C Tricco AC Pollock D Munn Z Alexander L . Updated methodological guidance for the conduct of scoping reviews. JBI Evidence Synthesis. (2020) 18:2119–26. doi: 10.11124/JBIES-20-00167, 33038124

[43] Pollock D Evans C Menghao Jia R Alexander L Pieper D Brandão de Moraes É . “How-to”: scoping review? J Clin Epidemiol. (2024) 176:111572. doi: 10.1016/j.jclinepi.2024.111572, 39426499

[44] Mak S Thomas A. Steps for conducting a scoping review. J Grad Med Educ. (2022) 14:565–7. doi: 10.4300/JGME-D-22-00621.1, 36274762 PMC9580325

[45] Demiris G Oliver DP Washington KT. Chapter 3 - defining and analyzing the problem In: Demiris G Oliver DP Washington KT, editors. Behavioral intervention research in hospice and palliative care: Academic Press (2019). 27–39.

[46] Sukhera J. Narrative reviews: flexible, rigorous, and practical. J Grad Med Educ. (2022) 14:414–7. doi: 10.4300/JGME-D-22-00480.1, 35991099 PMC9380636

[47] Chiew K-L Sundaresan P Jalaludin B Vinod SK. A narrative synthesis of the quality of cancer care and development of an integrated conceptual framework. Eur J Cancer Care. (2018) 27:e12881. doi: 10.1111/ecc.12881, 30028054

[48] Kitson A Marshall A Bassett K Zeitz K. What are the core elements of patient-centred care? A narrative review and synthesis of the literature from health policy, medicine and nursing. J Adv Nurs. (2013) 69:4–15. doi: 10.1111/j.1365-2648.2012.06064.x, 22709336

[49] McGreevy C Williams D. New insights about vitamin D and cardiovascular disease. Ann Intern Med. (2011) 155:820–6. doi: 10.7326/0003-4819-155-12-201112200-00004, 22184689

[50] Schwartz R Sinskey JL Anand U Margolis RD. Addressing Postpandemic clinician mental health. Ann Intern Med. (2020) 173:981–8. doi: 10.7326/M20-4199, 32822206 PMC7450528

[51] Willis VC Arriaga Y Weeraratne D Reyes F Jackson GP. A narrative review of emerging therapeutics for COVID-19. Mayo Clin Proc Innov Qual Outcomes. (2020) 4:745–58. doi: 10.1016/j.mayocpiqo.2020.07.004, 32838206 PMC7369591

[52] Briggs D. 1999 ‘Environmental health indicators: framework and methodologies’, World Health Organization. Protection of the human environment occupational and environmental health series Geneva, Switzerland: World Health Organization.

[53] Schwartz E Corvalán C. Decision-making in environmental health. London, UK: E & Fl\T Spon. (2000). 8585235

[54] Chiabai A Quiroga S Martinez-Juarez P Higgins S Taylor T. The nexus between climate change, ecosystem services and human health: towards a conceptual framework. Sci Total Environ. (2018) 635:1191–204. doi: 10.1016/j.scitotenv.2018.03.323, 29710574

[55] Gentry-Shields J Bartram J. Human health and the water environment: using the DPSEEA framework to identify the driving forces of disease. Sci Total Environ. (2014) 468-469:306–14. doi: 10.1016/j.scitotenv.2013.08.052, 24036221

[56] Salgueiro MF Fernández Salgueiro M Cernuda Martínez JA Gan RK Arcos González P. Climate change and antibiotic resistance: a scoping review. Environ Microbiol Rep. (2024) 16:e70008. doi: 10.1111/1758-2229.70008, 39267332 PMC11393301

[57] Anikeeva O Hansen A Varghese B Borg M Zhang Y Xiang J . The impact of increasing temperatures due to climate change on infectious diseases. BMJ. (2024) 387:e079343. doi: 10.1136/bmj-2024-079343, 39366706

[58] Hellberg RS Chu E. Effects of climate change on the persistence and dispersal of foodborne bacterial pathogens in the outdoor environment: A review. Crit Rev Microbiol. (2016) 42:548–72. doi: 10.3109/1040841X.2014.972335, 25612827

[59] Lindquist S. The heat-shock response. Annu Rev Biochem. (1986) 55:1151–91. doi: 10.1146/annurev.bi.55.070186.005443, 2427013

[60] Richter K Haslbeck M Buchner J. The heat shock response: life on the verge of death. Mol Cell. (2010) 40:253–66. doi: 10.1016/j.molcel.2010.10.006, 20965420

[61] Rodríguez-Verdugo A Gaut BS Tenaillon O. Evolution of *Escherichia coli* rifampicin resistance in an antibiotic-free environment during thermal stress. BMC Evol Biol. (2013) 13:50. doi: 10.1186/1471-2148-13-50, 23433244 PMC3598500

[62] Van Eldijk TJB Sheridan EA Martin G Weissing FJ Kuipers OP Van Doorn GS. Temperature dependence of the mutation rate towards antibiotic resistance. JAC Antimicrob Resist. (2024) 6:dlae085. doi: 10.1093/jacamr/dlae085, 38847007 PMC11154133

[63] Yang JW Nam JH Lee KJ Yoo JS. Effect of temperature on Carbapenemase-encoding plasmid transfer in *Klebsiella pneumoniae*. Microorganisms. (2024) 12:454. doi: 10.3390/microorganisms12030454, 38543505 PMC10972239

[64] Hashimoto M Hasegawa H Maeda S. High temperatures promote cell-to-cell plasmid transformation in *Escherichia coli*. Biochem Biophys Res Commun. (2019) 515:196–200. doi: 10.1016/j.bbrc.2019.05.134, 31138439

[65] Zhao W Zheng S Ye C Li J Yu X. Nonlinear impacts of temperature on antibiotic resistance in *Escherichia coli*. Environ. Sci Ecotechnol. (2024) 22:100475. doi: 10.1016/j.ese.2024.100475, 39280591 PMC11402153

[66] Kaba HEJ Kuhlmann E Scheithauer S. Thinking outside the box: association of antimicrobial resistance with climate warming in Europe – A 30 country observational study. Int J Hyg Environ Health. (2020) 223:151–8. doi: 10.1016/j.ijheh.2019.09.008, 31648934

[67] Pärnänen KMM Narciso-da-Rocha C Kneis D Berendonk TU Cacace D Do TT . Antibiotic resistance in European wastewater treatment plants mirrors the pattern of clinical antibiotic resistance prevalence. Sci Adv. (2019) 5:eaau9124. doi: 10.1126/sciadv.aau9124, 30944853 PMC6436925

[68] Li W Liu C Ho HC Shi L Zeng Y Yang X . Association between antibiotic resistance and increasing ambient temperature in China: an ecological study with nationwide panel data. Lancet Regional Health Western Pacific. (2023) 30:100628. doi: 10.1016/j.lanwpc.2022.100628, 36406382 PMC9672962

[69] Ahmed W Zhang Q Lobos A Senkbeil J Sadowsky MJ Harwood VJ . Precipitation influences pathogenic bacteria and antibiotic resistance gene abundance in storm drain outfalls in coastal sub-tropical waters. Environ Int. (2018) 116:308–18. doi: 10.1016/j.envint.2018.04.005, 29754026

[70] Ahmed W Gyawali P Hamilton KA Joshi S Aster D Donner E . Antibiotic resistance and sewage-associated marker genes in untreated sewage and a river characterized during baseflow and stormflow. Front Microbiol. (2021) 12:632850. doi: 10.3389/fmicb.2021.632850 34177821 PMC8226142

[71] Carney RL Labbate M Siboni N Tagg KA Mitrovic SM Seymour JR. Urban beaches are environmental hotspots for antibiotic resistance following rainfall. Water Res. (2019) 167:115081. doi: 10.1016/j.watres.2019.115081, 31574348

[72] Jang J Kim M Baek S Shin J Shin J Shin SG . Hydrometeorological influence on antibiotic-resistance genes (ARGs) and bacterial Community at a Recreational Beach in Korea. J Hazard Mater. (2021) 403:123599. doi: 10.1016/j.jhazmat.2020.123599, 32791479

[73] Stange C Tiehm A. Occurrence of antibiotic resistance genes and microbial source tracking markers in the water of a karst spring in Germany. Sci Total Environ. (2020) 742:140529. doi: 10.1016/j.scitotenv.2020.140529, 32629259

[74] Tipper HJ Stanton IC Payne RA Read DS Singer AC. Do storm overflows influence AMR in the environment and is this relevant to human health? A UK perspective on a global issue. Water Res. (2024) 260:121952. doi: 10.1016/j.watres.2024.121952, 38906083

[75] Drozdova J Raclavska H Skrobankova H. A survey of heavy metals in municipal wastewater in combined sewer systems during wet and dry weather periods. Urban Water J. (2015) 12:131–144. doi: 10.1080/1573062X.2013.831913

[76] Robas M Probanza A González D Jiménez PA. Mercury and antibiotic resistance co-selection in Bacillus sp. isolates from the Almadén Mining District. Int J Environ Res Public Health. (2021) 18:8304. doi: 10.3390/ijerph18168304, 34444052 PMC8392408

[77] Zhang S Wang Y Song H Lu J Yuan Z Guo J. Copper nanoparticles and copper ions promote horizontal transfer of plasmid-mediated multi-antibiotic resistance genes across bacterial genera. Environ Int. (2019) 129:478–87. doi: 10.1016/j.envint.2019.05.054, 31158594

[78] Belete B Desye B Ambelu A Yenew C. Micropollutant removal efficiency of advanced wastewater treatment plants: A systematic review. Environ Health Insights. (2023) 17:11786302231195158. doi: 10.1177/11786302231195158, 37692976 PMC10492480

[79] Karbalaei S Hanachi P Walker TR Cole M. Occurrence, sources, human health impacts and mitigation of microplastic pollution. Environ Sci Pollut Res Int. (2018) 25:36046–63. doi: 10.1007/s11356-018-3508-7, 30382517

[80] Liu Y Liu W Yang X Wang J Lin H Yang Y. Microplastics are a hotspot for antibiotic resistance genes: Progress and perspective. Sci Total Environ. (2021) 773:145643. doi: 10.1016/j.scitotenv.2021.145643, 33940744

[81] Yang QE Lin Z Gan D Li M Liu X Zhou S . Microplastics mediates the spread of antimicrobial resistance plasmids via modulating conjugal gene expression. Environ Int. (2025) 195:109261. doi: 10.1016/j.envint.2025.109261, 39813956

[82] EPA (2025) Nonpoint Source: Agriculture | US EPA. Available online at: https://www.epa.gov/nps/nonpoint-source-agriculture (Accessed: 30 September 2025).

[83] Westrich JR Ebling AM Landing WM Joyner JL Kemp KM Griffin DW . Saharan dust nutrients promote Vibrio bloom formation in marine surface waters. Proc Natl Acad Sci. (2016) 113:5964–9. doi: 10.1073/pnas.1518080113, 27162369 PMC4889353

[84] Caldwell JM Lambrechts L Rose NH. The role of vector population variation and climate in Zika virus transmission patterns in Africa: a modelling study. Lancet Planetary Health. (2024) 8:e1020–9. doi: 10.1016/S2542-5196(24)00276-6, 39674192 PMC12352338

[85] Downs J Downs J Mesev V Chakraborty S. Climate-induced expansion of Lyme disease in east Central Ohio. Int J Environ Health Res. (2025) 35:2717–27. doi: 10.1080/09603123.2025.2456966, 39876742

[86] Segala FV Guido G Stroffolini G Masini L Cattaneo P Moro L . Insights into the ecological and climate crisis: emerging infections threatening human health. Acta Trop. (2025) 262:107531. doi: 10.1016/j.actatropica.2025.107531, 39837368

[87] Zavaleta-Monestel E Rojas-Chinchilla C Molina-Sojo P Murillo-Castro MF Rojas-Molina JP Martínez-Vargas E. Impact of climate change on the global dynamics of vector-borne infectious diseases: A narrative review. Cureus. (2025) 17:e77972. doi: 10.7759/cureus.77972, 39996198 PMC11849761

[88] Chase JM Knight TM. Drought-induced mosquito outbreaks in wetlands. Ecol Lett. (2003) 6:1017–24. doi: 10.1046/j.1461-0248.2003.00533.x

[89] Pai H-H Chen W-C Peng C-F. Isolation of bacteria with antibiotic resistance from household cockroaches (Periplaneta americana and *Blattella germanica*). Acta Trop. (2005) 93:259–65. doi: 10.1016/j.actatropica.2004.11.006, 15716054

[90] Wei N Lu J Dong Y Li S. Profiles of microbial community and antibiotic Resistome in wild tick species. mSystems. (2022) 7. doi: 10.1128/msystems.00037-22, 35913190 PMC9426550

[91] Hassan B Ijaz M Khan A Sands K Serfas GI Clayfield L . A role for arthropods as vectors of multidrug-resistant Enterobacterales in surgical site infections from South Asia. Nat Microbiol. (2021) 6:1259–70. doi: 10.1038/s41564-021-00965-1, 34580444

[92] Semenza JC. Cascading risks of waterborne diseases from climate change. Nat Immunol. (2020) 21:484–7. doi: 10.1038/s41590-020-0631-7, 32313241

[93] Van de Vuurst P Escobar LE. Climate change and infectious disease: a review of evidence and research trends. Infect Dis Poverty. (2023) 12:51. doi: 10.1186/s40249-023-01102-2, 37194092 PMC10186327

[94] Mora C McKenzie T Gaw IM Dean JM von Hammerstein H Knudson TA . Over half of known human pathogenic diseases can be aggravated by climate change. Nat Clim Chang. (2022) 12:869–75. doi: 10.1038/s41558-022-01426-1, 35968032 PMC9362357

[95] Fisman D Patrozou E Carmeli Y Perencevich E Tuite AR Mermel LA . Geographical variability in the likelihood of bloodstream infections due to gram-negative Bacteria: correlation with proximity to the equator and health care expenditure. PLoS One. (2014) 9:e114548. doi: 10.1371/journal.pone.0114548, 25521300 PMC4270641

[96] Eber MR Shardell M Schweizer ML Laxminarayan R Perencevich EN. Seasonal and temperature-associated increases in gram-negative bacterial bloodstream infections among hospitalized patients. PLoS One. (2011) 6:e25298. doi: 10.1371/journal.pone.0025298, 21966489 PMC3180381

[97] Yavarian J Shafiei-Jandaghi NZ Mokhtari-Azad T. Possible viral infections in flood disasters: a review considering 2019 spring floods in Iran. Iranian J Microbiol. (2019) 11:85–9. doi: 10.18502/ijm.v11i2.1066, 31341561 PMC6635310

[98] Chadsuthi S Chalvet-Monfray K Wiratsudakul A Modchang C. The effects of flooding and weather conditions on leptospirosis transmission in Thailand. Sci Rep. (2021) 11:1486. doi: 10.1038/s41598-020-79546-x, 33452273 PMC7810882

[99] Furlan JPR Sellera FP Lincopan N Debone D Miraglia SGEK Tavella RA. Catastrophic floods and antimicrobial resistance: interconnected threats with wide-ranging impacts. One Health. (2024) 19:100891. doi: 10.1016/j.onehlt.2024.100891, 39310088 PMC11415860

[100] Liu Z Ding G Zhang Y Xu X Liu Q Jiang B. Analysis of risk and burden of dysentery associated with floods from 2004 to 2010 in Nanning, China. Am J Trop Med Hyg. (2015) 93:925–30. doi: 10.4269/ajtmh.14-0825, 26416103 PMC4703250

[101] Wolfe M Kaur M Yates T Woodin M Lantagne D. A systematic review and Meta-analysis of the association between water, sanitation, and hygiene exposures and cholera in case–control studies. Am J Trop Med Hyg. (2018) 99:534–45. doi: 10.4269/ajtmh.17-0897, 29968551 PMC6090371

[102] Gamble J.L. 2016) Ch. 9: populations of concern, the impacts of climate change on human health in the United States: A scientific assessment. U.S. Global Change Research Program, Washington, DC, 247–286

[103] Rawat A Karlstrom J Ameha A Oulare M Omer MD Desta HH . The contribution of community health systems to resilience: case study of the response to the drought in Ethiopia. J Glob Health. (2022) 12. doi: 10.7189/jogh.12.14001, 36273279 PMC9588157

[104] Bollinger E Zubrod JP Lai FY Ahrens L Filker S Lorke A . Antibiotics as a silent driver of climate change? A case study investigating methane production in freshwater sediments. Ecotoxicol Environ Saf. (2021) 228:113025. doi: 10.1016/j.ecoenv.2021.113025, 34847437

[105] Hammer TJ Fierer N Hardwick B Simojoki A Slade E Taponen J . Treating cattle with antibiotics affects greenhouse gas emissions, and microbiota in dung and dung beetles. Proc R Soc Lond B Biol Sci. (2016) 283:20160150. doi: 10.1098/rspb.2016.0150, 27226475 PMC4892788

[106] Nappier SP Liguori K Ichida AM Stewart JR Jones KR. Antibiotic resistance in recreational waters: state of the science. Int J Environ Res Public Health. (2020) 17. doi: 10.3390/ijerph17218034, 33142796 PMC7663426

[107] Leonard AFC Zhang L Balfour AJ Garside R Gaze WH. Human recreational exposure to antibiotic resistant bacteria in coastal bathing waters. Environ Int. (2015) 82:92–100. doi: 10.1016/j.envint.2015.02.013, 25832996

[108] Leonard AFC Zhang L Balfour AJ Garside R Hawkey PM Murray AK . Exposure to and colonisation by antibiotic-resistant *E. coli* in UK coastal water users: environmental surveillance, exposure assessment, and epidemiological study (beach bum survey). Environ Int. (2018) 114:326–33. doi: 10.1016/j.envint.2017.11.003, 29343413

[109] Farrell ML Chueiri A O'Connor L Duane S Maguire M Miliotis G . Assessing the impact of recreational water use on carriage of antimicrobial resistant organisms. Sci Total Environ. (2023) 888:164201. doi: 10.1016/j.scitotenv.2023.164201, 37196970

[110] Rees EE Davidson J Fairbrother JM St Hilaire S Saab M McClure JT. Occurrence and antimicrobial resistance of *Escherichia coli* in oysters and mussels from Atlantic Canada. Foodborne Pathog Dis. (2015) 12:164–9. doi: 10.1089/fpd.2014.1840, 25551332

[111] Svanevik CS Norström M Lunestad BT Slettemeås JS Urdahl AM. From tide to table: A whole-year, coastal-wide surveillance of antimicrobial resistance in *Escherichia coli* from marine bivalves. Int J Food Microbiol. (2023) 407:110422. doi: 10.1016/j.ijfoodmicro.2023.110422, 37804775

[112] Wang F Sun R Hu H Duan G Meng L Qiao M. The overlap of soil and vegetable microbes drives the transfer of antibiotic resistance genes from manure-amended soil to vegetables. Sci Total Environ. (2022) 828:154463. doi: 10.1016/j.scitotenv.2022.154463, 35276164

[113] Kläui A Bütikofer U Naskova J Wagner E Marti E. Fresh produce as a reservoir of antimicrobial resistance genes: A case study of Switzerland. Sci Total Environ. (2024) 907:167671. doi: 10.1016/j.scitotenv.2023.167671, 37813266

[114] Anand U Reddy B Singh VK Singh AK Kesari KK Tripathi P . Potential environmental and human health risks caused by antibiotic-resistant Bacteria (ARB), antibiotic resistance genes (ARGs) and emerging contaminants (ECs) from municipal solid waste (MSW) landfill. Antibiotics. (2021) 10:374. doi: 10.3390/antibiotics10040374, 33915892 PMC8065726

[115] Li S Ondon BS Ho SH Jiang J Li F. Antibiotic resistant bacteria and genes in wastewater treatment plants: from occurrence to treatment strategies. Sci Total Environ. (2022) 838:156544. doi: 10.1016/j.scitotenv.2022.156544, 35679932

[116] Morgado-Gamero WB Parody A Medina J Rodriguez-Villamizar LA Agudelo-Castañeda D. Multi-antibiotic resistant bacteria in landfill bioaerosols: environmental conditions and biological risk assessment. Environ Pollut. (2021) 290:118037. doi: 10.1016/j.envpol.2021.118037, 34482243

[117] Zhao Y Xiong M Ho K Rao Y Huang Y Cao J . Bioaerosol emission and exposure risk from a wastewater treatment plant in winter and spring. Ecotoxicol Environ Saf. (2024) 287:117294. doi: 10.1016/j.ecoenv.2024.117294, 39504877

[118] Berglund F Rodríguez-Molina D Gradisteanu Pircalabioru G Blaak H Chifiriuc MC Czobor Barbu I . The resistome and microbiome of wastewater treatment plant workers – the AWARE study. Environ Int. (2023) 180:108242. doi: 10.1016/j.envint.2023.108242, 37816267

[119] Magnusson U . Antimicrobial resistance in bacterial pathogens from farm animals In: Gross JJ, editor. Production diseases in farm animals: Pathophysiology, prophylaxis and health management. Cham: Springer International Publishing (2024). 25–46.

[120] Yang F Gao Y Zhao H Li J Cheng X Meng L . Revealing the distribution characteristics of antibiotic resistance genes and bacterial communities in animal-aerosol-human in a chicken farm: from one-health perspective. Ecotoxicol Environ Saf. (2021) 224:112687. doi: 10.1016/j.ecoenv.2021.112687, 34438267

[121] Fuhrmeister ER Harvey AP Nadimpalli ML Gallandat K Ambelu A Arnold BF . Evaluating the relationship between community water and sanitation access and the global burden of antibiotic resistance: an ecological study. Lancet Microbe. (2023) 4:e591–600. doi: 10.1016/S2666-5247(23)00137-4, 37399829 PMC10393780

[122] Hu Y Jiang L Sun X Wu J Ma L Zhou Y . Risk assessment of antibiotic resistance genes in the drinking water system. Sci Total Environ. (2021) 800:149650. doi: 10.1016/j.scitotenv.2021.149650, 34426368

[123] Noppert GA Hegde ST Kubale JT. Exposure, susceptibility, and recovery: A framework for examining the intersection of the social and physical environments and infectious disease risk. Am J Epidemiol. (2022) 192:475–82. doi: 10.1093/aje/kwac186, 36255177 PMC10372867

[124] Balasegaram M. Global trends and projections in antimicrobial resistance. Lancet. (2025) 405:1904–5. doi: 10.1016/S0140-6736(25)00806-2, 40449964

[125] Enesco A. ESCMID global 2025: three million child deaths tied to AMR in 2022. Eur Med J. (2025) Available online at: https://www.emjreviews.com/microbiology-infectious-diseases/news/escmid-global-2025-three-million-child-deaths-tied-to-amr-in-2022/. (Accessed 03 June, 2025).

[126] Hu YJ Qiu H Harwell JI Bryant P. Global geographic patterns and trends of WHO priority pathogens and AWaRe antibiotic resistance among children: an epidemiological surveillance study. Soc Sci Res Netw. (2023) doi: 10.2139/ssrn.4580795

[127] Augustine S Bonomo RA. Taking stock of infections and antibiotic resistance in the elderly and long-term care facilities: A survey of existingand upcoming challenges. European J Microbiol Immunol. (2011) 1:190–7. doi: 10.1556/EuJMI.1.2011.3.2, 24516724 PMC3906614

[128] Theodorakis N Feretzakis G Hitas C Kreouzi M Kalantzi S Spyridaki A . Antibiotic resistance in the elderly: mechanisms, risk factors, and solutions. Microorganisms. (2024) 12:1978. doi: 10.3390/microorganisms12101978, 39458286 PMC11509523

[129] Kwon JH Wi CI Seol HY Park M King K Ryu E . Risk, mechanisms and implications of asthma-associated infectious and inflammatory multimorbidities (AIMs) among individuals with asthma: a systematic review and a case study. Allergy, Asthma Immunol Res. (2021) 13:697–718. doi: 10.4168/aair.2021.13.5.697, 34486256 PMC8419637

[130] Carrillo-Larco RM Anza-Ramírez C Saal-Zapata G Villarreal-Zegarra D Zafra-Tanaka JH Ugarte-Gil C . Type 2 diabetes mellitus and antibiotic-resistant infections: a systematic review and meta-analysis. J Epidemiol Community Health. (2022) 76:75–84. doi: 10.1136/jech-2020-216029, 34326183 PMC8666814

[131] Kim EJ Ha KH Kim DJ Choi YH. Diabetes and the risk of infection: A National Cohort Study. Diabetes Metab J. (2019) 43:804–14. doi: 10.4093/dmj.2019.0071, 31701687 PMC6943267

[132] Alividza V Mariano V Ahmad R Charani E Rawson TM Holmes AH . Investigating the impact of poverty on colonization and infection with drug-resistant organisms in humans: a systematic review. Infect Dis Poverty. (2018) 7:76. doi: 10.1186/s40249-018-0459-7, 30115132 PMC6097281

[133] Allel K García P Labarca J Munita JM Rendic M Grupo Colaborativo de Resistencia Bacteriana . Socioeconomic factors associated with antimicrobial resistance of *Pseudomonas aeruginosa*, Staphylococcus aureus, and *Escherichia coli* in Chilean hospitals (2008–2017). Rev Panam Salud Publica. (2020) 44:1. doi: 10.26633/RPSP.2020.30, 32973892 PMC7498296

[134] Cooper LN Beauchamp AM Ingle TA Diaz MI Wakene AD Katterpalli C . Socioeconomic disparities and the prevalence of antimicrobial resistance. Clin Infect Dis. (2024) 79:1346–53. doi: 10.1093/cid/ciae313, 38845562 PMC11650857

[135] See I Wesson P Gualandi N Dumyati G Harrison LH Lesher L . Socioeconomic factors explain racial disparities in invasive community-associated methicillin-resistant *Staphylococcus aureus* disease rates. Clin Infect Dis. (2017) 64:597–604. doi: 10.1093/cid/ciw808, 28362911 PMC5656382

[136] Wu S Tannous E Haldane V Ellen ME Wei X. Barriers and facilitators of implementing interventions to improve appropriate antibiotic use in low- and middle-income countries: a systematic review based on the consolidated framework for implementation research. Implemen Sci. (2022) 17:30. doi: 10.1186/s13012-022-01209-4, 35550169 PMC9096759

[137] Artzy-Randrup Y Alonso D Pascual M. Transmission intensity and drug resistance in malaria population dynamics: implications for climate change. PLoS One. (2010) 5:e13588. doi: 10.1371/journal.pone.0013588, 21060886 PMC2965653

[138] Deeb R Tufford D Scott GI Moore JG Dow K. Impact of climate change on *Vibrio vulnificus* abundance and exposure risk. Estuaries and coasts: J Estuarine Res Federation. (2018) 41:2289–303. doi: 10.1007/s12237-018-0424-5, 31263385 PMC6602088

[139] Charalampous P Gorasso V Plass D Pires SM von der Lippe E Mereke A . Burden of non-communicable disease studies in Europe: a systematic review of data sources and methodological choices. Eur J Pub Health. (2022) 32:289–96. doi: 10.1093/eurpub/ckab218, 35015851 PMC8975530

[140] Vos T Lim SS Abbafati C Abbas KM Abbasi M Abbasifard M . Global burden of 369 diseases and injuries in 204 countries and territories, 1990–2019: a systematic analysis for the global burden of disease study 2019. Lancet. (2020) 396:1204–22. doi: 10.1016/S0140-6736(20)30925-9, 33069326 PMC7567026

[141] The Lancet Healthy Longevity. Tackling antimicrobial resistance to protect healthy ageing. Lancet Healthy Longevity. (2023) 4:e584. doi: 10.1016/S2666-7568(23)00218-0, 37924836

[142] WHO (2014) WHO’s first global report on antibiotic resistance reveals serious, worldwide threat to public health. Available online at: https://www.who.int/southeastasia/news/detail/30-04-2014-who-s-first-global-report-on-antibiotic-resistance-reveals-serious-worldwide-threat-to-public-health (Accessed: 3 June 2025).

[143] Spivak ES Tobin J Hersh AL Lee AP. Greenhouse gas emissions due to unnecessary antibiotic prescriptions. Antimicrob Steward Healthc Epidemiol. (2024) 4:e114. doi: 10.1017/ash.2024.354, 39257428 PMC11384152

[144] Slobodiuk S Niven C Arthur G Thakur S Ercumen A. Does irrigation with treated and untreated wastewater increase antimicrobial resistance in soil and water: A systematic review. Int J Environ Res Public Health. (2021) 18:11046. doi: 10.3390/ijerph182111046, 34769568 PMC8583129

[145] O’Neill J. Review on Antimicrobial Resistance. Tackling Drug-Resistant Infections Globally: Final Report and Recommendations. Welcome Trust. UK Government. (2016) Available online at: https://www.biomerieuxconnection.com/wp-content/uploads/2018/04/Tackling-Drug-Resistant-Infections-Globally_-Final-Report-and-Recommendations.pdf (Accessed 10 November 2021).

