# Supplementary material for: An integrated framework for antimicrobial resistance: links with climate change and vulnerability
Source: Front Public Health. 2026 Feb 3;13:1679189. doi: 10.3389/fpubh.2025.1679189 (PMC12909482; doi:10.3389/fpubh.2025.1679189)
Supplement: Supplementary file 1 [file Table_1.docx]

Supplementary Material

This document provides a summary table (**Table A**) of the 68 studies included in the final selection of the literature review, which informed the results section of the manuscript. The bibliographic details of each study are provided in the subsequent pages. The table includes the following elements:

- **Framework element(s):** Indicates the component(s) of the mDPSEEA framework (Driver, Pressure, State, Exposure, Effect, Action) and/or the IPCC risk framework (Hazard, Vulnerability) to which the study contributes.
- **Study type:** Describes the nature of the study, including categories such as experimental, observational, modelling, or review.
- **Keywords:** Specifies the keywords provided in the
- **Data type:** Specifies the type of data sources (e.g., environmental samples, databases)
- **AMR/pathogen indicators:** Identifies the indicators used in the study, such as prevalence of infections, abundance of antibiotic resistance genes (ARGs), abundance/distribution of antibiotic resistance bacteria (ARB), etc.
- **Key findings:** Summarises the main results of the study.
- **Supporting evidence**: Details the specific evidence (quantitative or qualitative) underpinning the key findings.
- **Relevance for the framework:** Highlights the contribution of the study to the review questions (e.g., evidence of links, identification of research gaps, Recommendations for actions).
- **Reference:** Provides the citation of the reviewed study.

**Note:** Table A uses abbreviations to enhance readability and conserve space.

- Framework elements: Hazard (driver, pressure, and state) is abbreviated as **Hazard (d–p–s)**; Vulnerability (exposure-effect, susceptibility, and coping capacity) as **Vulnerability (e-e, s, cp)**.
- Study types: Experimental studies are denoted as **Ex**.; Review studies as **Re**.; Reports as **Rep**.; Observational studies as **Ob**.; Opinion papers as **Op**.; Modelling studies as **Mo**.; Multimethod studies as **MM**.

**Table A.** Summary of characteristics of the studies included in the final list reviewed.

| **Element(s) of the framework** | **Study type** | **Keywords** | **Data type** | **AMR/pathogen indicators** | **Key finding(s)** | **Evidence(s)** | **Relevance for the framework** | **Reference** |
| --- | --- | --- | --- | --- | --- | --- | --- | --- |
| Hazard  (*d-p-s)* | Ex. | Stormwater  Faecal indicator bacteria  Pathogens  Quantitative PCR  Health risks  MFQPCR | *Primary data from*:  Environmental water samples  *Secondary data from:*  Observational data (precipitation and sewage overflow) | Abundance of antibiotic resistance genes (ARGs) | Results from this study suggest increased levels of certain ARGs and sewage-associated marker genes in stormflow river water samples compared to base flow conditions. | Significant differences were identified in *Escherichia coli* , ARG, intl1, and marker genes associated with wastewater, and by flow conditions (i.e., base versus storm) and site types (peri-urban versus urban) combined (R = 0.3668, p = 0.0001), where percentage differences between multifactorial groups ranged from 20.8% to 11.2%. | *Evidence(s) of link:*  Precipitation events and prevalence of AMR in the environment, in particular due to sewage overflows. | (Ahmed et al. 2021) |
| Hazard  (*d-p-s)* | Ex. | Microbial source tracking  Antibiotic resistance  Stormwater  Human health risks  Sewage pollution | *Primary data from:*  Environmental water samples  *Secondary data from:* observational data (precipitation and sewage overflow) | Detection and abundance of ARGs | Results from the study suggest that precipitation influences pathogenic bacteria and ARG abundance in storm drain outfalls. | The bla_KPC_ gene associated with carbapenem-resistant Enterobacteriaceae and the beta-lactam resistant gene (bla_NPS_) were only detected in wet weather samples. The frequency of integron genes Intl2 and Intl3 detection increased by 42% in wet weather samples. | *Evidence(s) of link:*  Precipitation events and prevalence of AMR in the environment, in particular due to sewage overflows. | (Ahmed et al. 2018) |
| Hazard  (*d-p-s)* | Ex. | Antibiotic resistance  Storm-water Sewage  Microbial ecology  Quantitative PCR  Beach | *Primary data from:*  Environmental water samples  *Secondary data from:*  Observational data (precipitation and sewage overflow) | Detection and abundance of ARGs | The results suggest a link between stormwater discharge and sewer overflow events, and the occurrence of ARGs on coastal beaches. | Up to 100-fold increases in the abundance of several ARGs, including genes conferring resistance to quinolones, trimethoprim, sulfonamides, tetracycline, vancomycin and carbapenems, occurred following stormwater and modelled wet-weather sewer overflow events. | *Evidence(s) of link:*  Precipitation events and prevalence of AMR in the environment, in particular due to sewage overflows. | (Carney et al. 2019) |
| Hazard  (*d-p-s)*  *Action* | Ex. | Antibiotic-resistance genes  Microbial community  Combined sewage overflow  Rainfall event  Coastal water quality | *Primary data from:*  Environmental seawater samples  *Secondary data from:* Observational data (precipitation and sewage overflow) | Occurrence and distribution of ARGs | The results suggest a link between combined sewer overflow and sewer overflow events and the occurrence of ARGs in coastal recreational areas. | The result showed an influence of rainfall and tidal levels on the relative abundance of total ARGs and bacterial operational taxonomic units (OTUs), which showed 1.9 × 103 and 1.1 × 101 fold increases, respectively. In particular, the elevated levels of ARGs were maintained for up to 32 hours after rainfall. | *Evidence(s) of link:*  Precipitation events and prevalence of AMR in the environment, in particular due to sewage overflows.  *Recommendation(s) for action:*  Long-term monitoring of coastal recreational areas is required, especially after rainfall. | (Jang et al. 2021) |
| Hazard  (*d-p-s)* | Ex. | Karst spring  Spring water  Faecal indicator bacteria Antibiotic resistance genes  Microbial source tracking | *Primary data from:*  environmental water samples  *Secondary data from:* Observational data (precipitation and sewage overflow) | Occurrence of ARGs, ARG-associated faecal indicator bacteria (FIB) abundance | The results suggest links between heavy precipitation, overflow of the combined sewer in the basin and an increase in bacteria and ARGs in the water. | The ARGs most frequently detected were ermB (42.1%), tet(C) (40.8%), sul2 (39.5%), and sul1 (36.8%), which code for resistance to macrolides, tetracycline and sulfonamides, respectively. After a heavy rain event, the increase in FIB in the spring water was associated with the increase in ARGs and human-specific microbial source tracking (MST) markers. | *Evidence(s) of link:*  Precipitation events and prevalence of AMR in the environment, in particular due to sewage overflows. | (Stange and Tiehm 2020) |
| Hazard  (*d-p-s)*  Action | Re. | Antimicrobial resistance (AMR)  Water quality  Wastewater  Combined sewer overflows (CSOS)  One Health | *Secondary data from*:  Reviewed literature, observational data (sewage overflow) | Mixed indicators such as abundance of ARGs and FIB | The review describes global data investigating the impact of storm overflows (SOs) on environmental AMR, and discusses the implications of SOs regarding AMR and human health.  It also identifies some specific implications for the UK context. | The review provided a summary table with the results on the global literature on AMR and SOs, including quantitative and qualitative evidence. | *Evidence(s) of link:*  Precipitation events and prevalence of AMR in the environment, in particular due to sewage overflows.  *Knowledge gap(s):*  Limited evidence on AMR in/from SOs  (non-existence for the UK at the time of the review); lack of understanding of the sources (point and diffuse) of the pollution as mixed in the river at the catchment scale, and lack of evidence on the health impacts of environmental AMR.  *Recommendation(s) for action:* availability of real-time spill data, along with proper reporting mechanisms and regulatory frameworks for AMR surveillance in the environment and wastewater | (Tipper et al. 2024) |
| Hazard  (*d-p-s)* | Ex. | Coastal area  Combined sewer overflow  E. coli  Enterococci  Wastewater management and treatment  Wastewater treatment plant  effluent | *Primary data from:*  Environmental water samples  *Secondary data from:* Observational data (precipitation and sewage overflow) | FIB abundance | Combined sewer overflow (CSOs) is a main cause of microbial discharge load in coastal waters. | It was also found that the modest water volume discharged by all CSO outfalls (only 8% of the total volume discharged by the area) contains > 90% of the microbial load | *Evidence(s) of link:*  Precipitation events and prevalence of AMR in the environment, in particular due to sewage overflows. | (Al Aukidy and Verlicchi 2017) |
| Hazard  (*d-p-s)* | Ex. | CA; dry weather flow; heavy metals; municipal wastewater; PCA; wet weather flow | *Primary data from:*  Environmental water samples | NA | Heavy metals | During wet weather periods, concentrations of As, Cr, Cd, Pb, Mn, and Fe increased in effluent samples. By contrast, concentrations of Zn, Cu, and Ni decreased under the same conditions, while Hg levels did not change significantly. | *Evidence(s) of link:*  Heterogeneity of heavy metals in sewage overflows | (Drozdova et al. 2015) |
| Hazard  (*d-p-s)* | Ex. | *E. coli*  Horizontal gene transfer (HGT)  Transformation Temperature | *Primary data from:*  Laboratory experiment (*E. coli* exposed to different temperatures) | (HPT) Cell-to-cell plasmid transformation frequency | It was found that high temperatures of approximately 41-45ºC significantly promote cell-to-cell plasmid transformation in *E. coli*. | HPT occurred, to a greater or lesser extent, over a wide temperature range between 10-49 ºC, and high HPT frequency above 2 x 10^-8/^cell was observed between 37-47 ºC, with the highest frequency (~6 x10^8^/cell) occurring at 43-45 ºC. Temperatures of 41-43ºC produced the highest frequency (~9x 10^9^/cell) in liquid culture. HPT frequency in solid-air (SA) biofilm culture was significantly higher than that in liquid culture at any temperatures. These results indicate that the enhancing effect of high temperature on HPT is maximal at around 41-45 °C, and that it is more frequent in SA biofilms. | *Evidence(s) of link:*  Warmer temperatures and HGT | (Hashimoto et al. 2019) |
| Hazard  (*d-p-s)* | Ex.l | Antibiotic resistance  Temperature  Gatifloxacin  Resistance evolution  Fitness costs | *Primary data from:*  Laboratory experiment (*E. coli* exposed to different temperatures and antibiotics) | Prevalence ARB, HGT, etc. | Antibiotic resistance exhibits a nonlinear response to elevated temperatures under the combined stress of temperature and antibiotics. | The effectiveness of gatifloxacin against *Escherichia coli* significantly diminishes at 42ºC, while resistance increases 256-fold at 27ºC. Additionally, the increased transcription levels of genes such as marA, ygfA, and ibpB are observed with rising temperatures. | *Evidence(s) of link:*  Complexity of bacterial responses to elevated temperature and antibiotics. | (W. Zhao et al. 2024) |
| Hazard  (*d-p-s)* | Ob. | Antibiotic resistance  *Carbapenemase-producing Enterobacterales*  *Klebsiella pneumoniae*  Plasmid transfer  Temperature | *Secondary data from:*  Observational data (Surveillance system in hospitals, temperature) | Conjugation efficiency of plasmids carrying carbapenem-resistance genes | The study findings suggest that seasons promote the transfer of more antibiotic resistance-related genes, highlighting the importance of local temperature in the spread and transmission of plasmids carrying carbapenemases. | The optimal conjugation temperatures demonstrating the highest stability for bla_KPC_- and bla_NDM_-carrying plasmids were 25ºC (p = 0.030) and 30ºC (p = 0.007), respectively. The stability of bla_KPC_-IncF was higher at 25ºC than that at 30ºC (p = 0.032) or 37ºC (p = 0.047), while bla_KPC_-IncX3 exhibited the lowest stability at 37ºC (p = 0.047). bla_NDM_-IncX3 was more stable at 30ºC than at 37ºC (p = 0.049). | *Evidence(s) of link:*  Warmer temperatures and HGT | (Yang et al. 2024) |
| Hazard  (*d-p-s)* | Ex. | NA | *Primary data from:*  Laboratory experiment (*E. coli* exposed to different temperatures) | Mutation rate | The mutation rate towards antibiotic resistance is impacted by a small change in temperature. | The mutation rate towards ciprofloxacin and rifampicin resistance was higher at 40°C than at 37°C. | *Evidence(s) of link:* Temperature and the novo antibiotic resistance mutations | (Van Eldijk et al. 2024) |
| Hazard  (*d-p-s)* | Re. | NA | *Secondary data from*:  Reviewed literature | Mutation rate, HGT, prevalence of ARB, and prevalence of infections. | Changes in temperatures are likely to lead to thermal adaptation in microbes that experience rises and declines in temperature—either inside a host or in the external environment. These thermal adaptations may have collateral effects such as higher antibiotic resistance. | The text includes supporting quantitative data and qualitative descriptions of the links between temperature changes and selection of antibiotic resistance. This review examines these links by synthesising results from laboratories, hospitals, and environmental studies. | *Evidence(s) of link:* Temperature could promote the selection of AMR, through the novo antibiotic resistance mutations  *Recommendation(s) for action:* there is a need for more interdisciplinary research, as these issues correspond to multiple specialty scales: genomic, cellular, physiological, ecological, and encompass enormous ranges in spatial and temporal scales. | (Rodríguez-Verdugo et al. 2020) |
| Hazard  (*d-p-s)* | Ex | Beneficial mutations  Fitness effects  Experimental evolution  Trade-offs  Pleiotropy  Epistasis | *Primary data from:*  Laboratory experiment (*E. coli* exposed to different temperatures) | Antibiotic resistance mutations | The study evidenced the selection and fixation of resistant mutations in populations of *E. coli B* that had never been exposed to antibiotics. | The results found parallel mutations within the rpoB gene encoding the beta subunit of RNA polymerase. These amino acid substitutions conferred different levels of rifampicin resistance. The resistant mutations typically appeared and were fixed early in the evolution experiment. The study confirmed the high advantage of these mutations at 42.2°C in glucose-limited medium. | *Evidence(s) of link:* temperature could promote AMR in the absence of antibiotics. | (Rodríguez-Verdugo et al. 2013) |
| Hazard  (*d-p-s)*  Action | Re. | NA | *Secondary data from*:  Reviewed literature, observational data (sewage overflow) | Distribution and abundance of vectors, prevalence of infectious diseases, pooled risk and infection transmission rate | The results from this review suggest there is an increase in the risk of infectious diseases due to higher temperatures.  It also provided a summary of key actions. | The review provided a summary table of the impacts of high temperature on selected infectious diseases, including quantitative and qualitative evidence.  Includes a summary illustration of the requirements for an integrated surveillance system for climate-sensitive infectious diseases. | *Evidence(s) of link:*  Warmer temperatures increase infectious disease risks.  *Recommendation(s) for action:* Strengthen integrated surveillance and early warning systems; use satellite remote sensing and GIS technologies to map infectious disease distribution and allocate resources to high-risk areas; support vulnerable regions; ensure quality of risk assessments and investigate how AI can improve infectious disease risk assessments; integrate climate-health education into health professionals’ training. | (Anikeeva et al. 2024) |
| Hazard  (*d-p-s)* | Ex.l | Insect  Microbiome  Antimicrobial  Methane  Greenhouse gases | *Primary data from:*  Dung and beetle microbiota samples, and greenhouse house emissions flux from dung. | NA | The results suggest that antibiotics restructure dung beetle microbiota and modify greenhouse gas emissions from dung, indicating that antibiotic treatment may have unintended, cascading ecological effects that extend beyond the target animal | Antibiotic treatment consistently increased methane emissions (F_1,36_ =22.21, p < 0.0001). | *Plausible feedback loop*: antibiotic treatment in cattle could contribute to greenhouse gas emissions | (Hammer et al. 2016) |
| Hazard  (*d-p-s)* | Ex. | Antibiotics  Methanogenesis  Freshwater sediments  Metabarcoding  Compound-specific isotope analysis  Climate change | *Primary data from: E*nvironmental sediment samples | NA | The results suggest that antibiotics can influence methane production in freshwater systems. | Results showed that the CH_4_ production rate was increased by up to 94% at the high antibiotic concentrations (5000 μg/L) and up to 29% at field-relevant concentrations (50 μg/L). | *Plausible feedback loop*: Antibiotics in the environment could contribute to greenhouse gas emissions | (Bollinger et al. 2021) |
| Hazard  (*d-p-s)* | Mo. | NA | *Secondary data from:*  Observational data (Surveillance system in hospitals, temperature) | Incidence rate ratio of infections due to Gram-negative bacteria (GNB) | The likelihood of bacteremia due to Gram-negative bacteria varies markedly between cities, in a manner that appears to have both geographic (latitude) and socioeconomic (proportion gross domestic product devoted to health spending) determinants. | In multivariable models, only the percentage of gross domestic product spent on healthcare and latitude-squared (i.e., distance from the equator) were associated with the fraction of bloodstream infections due to Gram-negative bacteria at the P<0.05. | *Evidence(s) of link:*  Warmer temperatures and infectious disease risks  Socioeconomic factors influence the risk of infections. | (Fisman et al. 2014) |
| Hazard  (*d-p-s)* | Mo. | NA | *Secondary data from:*  Observational data (Surveillance system in hospitals, temperature) | BSI (bloodstream infections) counts | Summer season and higher mean monthly outdoor temperature are associated with substantially increased frequency of BSIs, particularly among clinically important Gram-negative bacteria. | An increase in mean monthly temperature of 5.6ºC (10ºF) corresponded to independent increases in *Acinetobacter, E. coli,* *K. pneumonia, and P. aeruginosa* BSI frequencies of 10.8% (95% CI 6.9–14.7), 3.5% (95% CI 2.1–4.9), 8.0% (95% CI 6.0–10.1), and 7.5% (95% CI 5.1–10.0), respectively. For *S. aureus*, an increase in temperature of 5.6ºC (10ºF) was associated with an adjusted increase of 2.2% (95% CI 1.3–3.2). | *Evidence(s) of link:*  Warmer temperatures increase infectious disease risks | (Eber et al. 2011) |
| Hazard  (*d-p-s)* | Mo. | Antibiotic resistance  Ambient temperature  Climate change  China | *Secondary data from:*  Observational data (Surveillance system in hospitals, temperature, socioeconomic data) | Antibiotic-resistant infections | Higher prevalence of antibiotic resistance is associated with increased regional ambient temperature. | 1◦C increase in average ambient temperature was associated with a 1.14-fold increase (95%-CI [1.07–1.23]) in CRKP prevalence and 1.06-fold increase (95%-CI [1.03–1.08]) in CRPA prevalence. There was an accumulative effect of year-by-year changes in ambient temperature, with the four-year sum showing the greatest effect on antibiotic resistance. Higher prevalence of antibiotic resistance was also associated with higher antibiotic consumption, lower density of health facilities, higher density of hospital beds and higher level of corruption. | *Evidence(s) of link:*  Warmer temperatures increase antibiotic resistance | (Li et al. 2023) |
| Hazard  (*d-p-s)* | Mo. | NA | *Secondary data from:*  Observational data (Surveillance system in hospitals, temperature) | Normalised antibiotic resistance | Associations between temperature and antibiotic resistance in this ecological study are consistent across most classes of antibiotics and pathogens and may be strengthening over time. | A 10°C increase in minimum temperature across the regions was associated with increases in antibiotic resistance of 4.2% (P < 0.0001), 2.2% (P < 0.0001), and 2.7% (P = 0.21) for *E. coli*, *K. pneumoniae*, and *S. aureus*, respectively. | *Evidence(s) of link:*  Warmer temperatures increase antibiotic resistance | (MacFadden et al. 2018) |
| Hazard  (*d-p-s)* | Mo. | Antibiotic resistance  Temperature Europe | *Secondary data from:*  Observational data (Surveillance systems in hospitals, temperature) | Normalised antibiotic resistance | Long-term effect of ambient minimum temperature on antibiotic resistance rate increases in Europe. Ambient temperature might considerably influence antibiotic resistance growth rates, and explain geographic differences observed in cross-sectional studies. Rising temperatures globally may hasten resistance spread, complicating mitigation efforts. | Results: During 2000–2016, for *Escherichia coli* and *Klebsiella pneumoniae*, European countries with 10°C warmer ambient minimum temperatures compared to others, experienced more rapid resistance increases across all antibiotic classes. Increases ranged between 0.33%/year (95% CI: 0.2 to 0.5) and 1.2%/year (95% CI: 0.4 to 1.9), even after accounting for recognised resistance drivers including antibiotic consumption and population density. For *Staphylococcus aureus* a decreasing relationship of −0.4%/year (95% CI: −0.7 to 0.0) was found for meticillin resistance, reflecting widespread declines in methicillin-resistant *S. aureus* across Europe over the study period. | *Evidence(s) of link:*  Warmer temperatures and antibiotic resistance | (McGough et al. 2020) |
| Hazard  (*d-p-s)* | Mo. | Antimicrobial resistance  P. aeruginosa  European countries  Climate warming  Log-linear reg | *Secondary data from:*  Observational data (Surveillance, temperature) | Antibiotic-resistant infections | Cimatic factors significantly contribute to the explanation of AMR in different types of healthcare systems, while climate change (i.e. warming) might increase AMR transmission, in particular CRPA. | A cross-sectional study across 30 European countries reported that a 0.5 °C increase in annual temperature change (exposure) was associated with a 1.02-fold increase (p = 0.035) in the prevalence of carbapenem-resistant *Pseudomonas aeruginosa* (CRPA). oreover, we found significant associations of CRKP, MREC, and MRSA with the warmseason mean temperature, which had a higher contribution to MRSA variance explanation than outpatient antimicrobial drug use. | *Evidence(s) of link:*  Warmer temperatures and antibiotic resistance | (Kaba et al. 2020) |
| Hazard  (*d-p-s)* | MM | NA | *Primary data from:*  Wastewater samples  *Secondary data from:*  Observational data (latitude, clinical isolates) | ARGs abundance | The first trans-European surveillance showed that urban wastewater treatment plants (UWTP) antibiotic resistance (AR) profiles mirror the AR gradient observed in clinics. Antibiotic use, environmental temperature, and UWTP size were important factors related to resistance persistence and spread in the environment. | However, the ARG burden after wastewater treatment was significantly higher in the south (H_AC_) than in the north (L_AC_) countries. As a consequence, it is hypothesised that the final effluents of the HAC countries may have a higher impact on the receiving environment than those of the L_AC_ countries. | *Evidence(s) of link(s):*  Warmer temperatures and antibiotic resistance  *Recommendation(s) for action(s):*  The use of traditional faecal indicators may have a limited capacity to provide reliable comparisons of antibiotic resistance status in wastewaters; long-term monitoring of WWTPs | (Pärnänen et al. 2019) |
| Hazard  (*d-p-s)* | Ex. | Bacillus  antibiotic resistance  mercury resistance  resistance co-selection  soil contamination | *Primary data from:*  Laboratory experiment: | Prevalence ARB | Environmental co-selection of antibiotic resistance AR to cephalosporins and tetracyclines by the selective pressure of Hg has been statistically demonstrated. | A total of 72% of *Bacillus spp*. showed resistance to two or more commonly used antibiotics. A total of 38 isolates expressed AR to cephalosporins. | *Evidence(s) of link(s):*  Heavy metals and antibiotic resistance | (Robas et al. 2021) |
| Hazard  (*d-p-s)* | Ex. | Copper oxide nanoparticles  Copper ions  Antibiotic resistance genes  Horizontal gene transfer  Conjugative transfer  Genome-wide RNA sequencing | Laboratory experiment:  (E. coli, plasmid donor, and *Pseudomonas putida*, exposed to to CuO NPs or Cu^2+^) | Plasmid transfer rate | Heavy metals can promote the HGT frequency of ARGs | When exposing bacteria to CuO NPs or Cu^2+^ at environmentally relevant and sub-inhibitory concentrations (e.g., 1–100 μmol/L), conjugation frequencies of plasmid-encoded antibiotic resistance genes across genera (i.e., from *Escherichia coli* to *Pseudomonas putida*) were significantly enhanced (p < 0.05). | *Evidence(s) of link(s):*  Heavy metals and antibiotic resistance | (Zhang et al. 2019) |
| Hazard  (*d-p-s)*  *E-E* | Ex. | Longitudinal monitoring  Airborne bacteria  Antibiotic resistomes  Exposure  *Staphylococcus* | *Primary data from:*  Airborne samples | Relative bacterial abundance, relative ARGs abundance | Compared with winter, P1 exposed a greater diversity of airborne bacteria in spring.  An extremely high relative abundance of ARGs (lunA/qacG) was found in the samples.  *Staphylococcu*s was a major bacterial host for ARGs in the personal airborne exposome. | The diversity of P1's microbial exposure communities was significantly greater in spring (P < 0.05) than those collected in winter. Similar to the microbiome, the alpha diversity and ARG diversity for P1 were significantly greater in spring (P < 0.05) than in summer and winter.  Two ARG subtypes, lnuA (MLS resistance) and qacG (multidrug resistance), accounted for a high prevalence of 30.83% and 29.07 % in all samples.  Samples from P1 and P2 possessed the highest prevalence of Staphylococcus spp. (40.30 %, 81/ 201 and 21.21 %, 10/33, respectively) and P3 samples possessed the highest prevalence of Bacillus spp. (41.67 %, 5/12). | *Evidence(s) of link(s):*  Warmer temperatures and diversity of airborne bacteria | (Zhang et al. 2023) |
| Hazard  (*d-p-s)* | Ex. | Antimicrobial resistance  Microplastics Plasmids  Horizontal gene transfer  Conjugation | *Primary data from:*  Laboratory experiment  (*E. coli,* plasmid donor, and *Klebsiella pneumoniae* recipients, exposed to different MPs and antibiotics) | Plasmid transfer rate | Exposure to commonly found MPs promotes the spread of AMR | Our study shows that exposure to four commonly found MPs promotes the conjugation rates of four clinically relevant AMR plasmids by up to 200-fold, when compared to the non-exposed group and that the transfer rates are MP concentrations demonstrate a positive correlation with higher transfer rates. Furthermore, we show that MPs induce the expression of plasmid-borne conjugal genes and SOS-linked genes such as recA, lexA, dinB and dinD. | *Evidence(s) of link(s):*  MPs and the spread of AMR  *Recommendation(s) for action(s):*  To tackle global AMR, we must also now consider plastic utilisation and waste management | (Yang et al. 2025) |
| Hazard  (*d-p-s)* | MM | Saharan dust  *Vibrio*  Iron  Marine Biogeochemistry  Microbial ecology | *Primary data from:*  Wastewater and sand samples  *Secondary data from:*  observational data (Saharan dust events) | Bacterial growth | Findings showed that *Vibrio* proliferate in response to a broad range of dust-Fe additions at rapid timescales. | Within 24 h of exposure, strains of *Vibrio cholerae* and *Vibrio alginolyticus* were able to directly use Saharan dust–Fe to support rapid growth. These findings were also confirmed with in situ field studies; arrival of Saharan dust in the Caribbean and subtropical Atlantic coincided with high levels of dissolved Fe, followed by up to a 30-fold increase of culturable *Vibrio* over background levels within 24 h. The relative abundance of Vibrio increased from ∼1to∼20% of the total microbial community. | *Evidence(s) of link(s):*  Dust nutrients in water and bacterial growth | (Westrich et al. 2016) |
| Hazard  (*d-p-s)*  *E-E* | Mo. | NA | *Secondary data from:*  Observational (climate data and surveillance) | Transmission rate | Flooding strongly contributes to disease transmission, where a high degree of flooding leads to a higher number of infected individuals. | Sensitivity analysis showed that the transmission rate of leptospires from a contaminated environment was the most important parameter for the total number of human cases. Our results suggest that public education should target people who work in contaminated environments to prevent Leptospira infections. | *Evidence(s) of link:*  Flooding and risk of infectious diseases | (Chadsuthi et al. 2021) |
| Hazard  (*d-p-s)* | Ex. | Competition  Drought  Food web  Mosquito  Outbreak  Precipitation  Predation. | *Primary data from:*  Environmental samples | Number of vectors (mosquitoes) | The study found that mosquito density increased dramatically following a natural drought event in a survey of wetlands. | Competitor biomass was highest in the temporary treatments, intermediate in the semi-permanent treatments and lowest in the permanent treatments (all Tukey’s HSD; P < 0.05) (Fig. 2b). Predator biomass was highest in the permanent treatments (Tukey’s HSD; P < 0.01), but equally low in both the temporary and semi-permanent treatments (Tukey’s HSD; P > 0.2). | *Evidence(s) of link:*  Drought and risk of infectious diseases | (Chase and Knight 2003) |
| Hazard  (*d-p-s)* | Mo. | Tick-borne illnesses  Climate change  Time series  Environmental health | *Secondary data from:*  Observational (climate data and surveillance) | Prevalence of infections | The study found that milder winters and increased spring and summer rainfall may further exacerbate Lyme disease cases in Ohio. | Results show that warmer winter temperatures, higher precipitation, and negative Southern Oscillation Index (SOI) values (El Niño conditions) were significantly associated with increased Lyme disease incidence and displayed delayed effects of 6 to18 months. | *Evidence(s) of link:*  Warmer temperatures and higher precipitation, and prevalence of infectious diseases prevalence | (Downs et al. 2025) |
| Hazard  (*d-p-s)*  *Action* | Op. | Climate change  Global warming Multidrug-resistant microorganisms Emerging pathogens Public health | *Secondary data from:*  Literature references | ARB, ARGs, colonization, infections | The opinion highlights the complex interplay between floods due to climate change and the spread of AMR. | The opinion provides an infographic that highlights the multiple environmental spread of AMR exacerbated by catastrophic floods. | *Evidence(s) of link:*  Flooding and risk of infectious diseases  *Recommendation(s) for action:* need to integrate climate resilience with AMR strategies should be discussed | (Furlan et al. 2024) |
| Hazard  (*d-p-s)* | Re. | Climate change  Food safety  Foodborne pathogens  Predictive models  zoonotic pathogens | *Secondary data from:*  Literature references | Persistence and dispersal of pathogens | The review article examines the effects of climatic factors, such as temperature, rainfall, drought and wind, on the environmental dispersal and persistence of bacterial foodborne pathogens, namely, *Bacillus cereus, Brucella, Campylobacter, Clostridium, Escherichia coli, Listeria monocytogenes, Salmonella, Staphylococcus aureus, Vibrio* and *Yersinia enterocolitica.* | The review offers a table summarising evidence of the effects of temperature and precipitation on the persistence and dispersal of foodborne pathogens in the environment. | *Recommendation(s) for action:*  Climate factors like wind and dust events are understudied but may become increasingly relevant. Significant research gaps remain, especially in quantifying climate–pathogen relationships. Predictive models and proactive food safety measures require interdisciplinary collaboration across climate, ecosystem, and food safety sciences. | (Hellberg and Chu 2016) |
| Hazard  (*d-p-s)* | Mo. | NA | *Secondary data from:*  Observational (climate data and surveillance) | Infections prevalence | The study confirms that floods have significantly increased the risk and the burden of dysentery in the study area. Public health action should be taken to prevent and control the potential risk of dysentery after floods. Vulnerable groups such as males and children should be paid more attention. | The relative risk (RR) of floods on the morbidity of dysentery was 1.44 (95% confidence interval [CI] = 1.18–1.75). The models suggest that a potential 1-day rise in flood duration may lead to 8% (RR = 1.08, 95% CI = 1.04–1.12) increase in the morbidity of dysentery. The average attributable YLD per 1,000 of dysentery caused by floods were 0.013 in males, 0.005 in females, and 0.009 in persons. | *Evidence(s) of link:*  Flooding and risk of infectious diseases | (Liu et al. 2015) |
| Hazard  (*d-p-s)* | Ex. | NA | *Primary data from:*  Samples from hospitals and arthropods | Prevalence of ARGS | The study showed the role for arthropods as vectors of multidrug resistant Enterobacterales in surgical site infections | The carbapenemase gene blaNDM was most commonly detected, with 15.5%, 15.1% and 13.3% of samples positive in SSIs, HSs and arthropods, respectively. SNP (≤20) and spatiotemporal analysis revealed linkages in bacteria between SSIs, HSs and arthropods supporting the One Health approach to underpin infection control policies across LMICs and control AMR | *Evidence(s) of link:* Arthropods as potential vectors of ARGs | (Hassan et al. 2021) |
| Hazard  (*d-p-s)* | Ex. | Wild ticks  Microbiome  Antibiotic resistance  Antibiotic resistance genes (ARGs)  Tick  Microbiome | *Secondary data from:*  Literature references | ARGs and mobile genetic elements (MGEs) | The study identifies themicrobial community and antibiotic resistome in wild tick species | In total, 100 different ARGs across 12 antibiotic classes and 20 mobile genetic elements (MGEs) were identified by HT-qPCR, and among them aminoglycosides, multidrug, macrolide-clinolamide-streptogramin B, and tetracycline resistance genes were the dominant ARG types | *Evidence(s) of link:*  Ticks as potential vectors of ARGs | (Wei et al. 2022) |
| Hazard  (*d-p-s)* | Ex. | Bacteria  Blattella germanica  Cockroaches  Antibiotic resistance  Vectors  Periplaneta americana | *Primary data from:*  Samples from household cockroaches gut | Prevalence of ARB | Potential role of cockroaches in the transmission of pathogenic bacteria with antibiotic resistance in households. | Cockroach infestation was found in 50% of the studied households and 226 cockroaches (123 P. americana and 103 B. germanica) collected by trapping. P. americana was more often found in the kitchen (70.7%) whereas B. germanica in the storage room (51.5%) and kitchen (36.9%). There was no significant difference between the percentages of P. americana (99.9%) and B. germanica (98.0%) carrying bacteria. A total of 25 species of bacteria was isolated from P. americana and only 21 from B. germanica. Antibiotic resistance was found in Staphylococcus aureus, Enterococcus species, Pseudomonas aeruginosa, Klebsiella pneumoniae, Escherichia coli, Serratia marcescens, and Proteus species isolated from the cockroaches. | *Evidence(s) of link:* Cockroaches as potential vectors of ARB | (Pai et al. 2005) |
| Hazard  (*d-p-s)* | Ex. | Microplastics (MPs)  Activated sludge Antibiotic resistance Sulfonamide Biofilm | *Primary data from:*  MPs samples from activated sludges | ARGs | Microplastics can serve as carriers of antibiotic-resistant bacteria (ARB) and pathogens, representing a pressing concern to aquatic biota and human health. | Both polyethylene (PE) and polystyrene (PS) microplastics can acclimate biofilms enriched with sulfonamide resistance genes (sul1 and sul2) and the associated mobile genetic element (intI1) in comparison with fine sands as control particles. Absolute abundances of these genes were further elevated by 1.2-4.5 fold when sulfamethoxazole was initially spiked as a representative sulfonamide. The combination of 16S rRNA amplicon sequencing and differential ranking analysis revealed that microplastics selectively promoted antibiotic-resistant and pathogenic taxa (e.g., Raoultella ornithinolytica and Stenotrophomonas maltophilia) with enrichment indices ranging 1.6 - 3.3. | *Evidence(s) of link:*  MPs and selection and spread of AMR | (Pham et al. 2021) |
| Vulnerability (e-e, s, cp) | Ex. | Beta-lactamase genes  IncC plasmids blaNDM-1  Recreational beaches  Mobile genetic elements | *Primary data from:*  Recreational water samples | Bacteria carrying clinically relevant ARGs associated with mobile genetic elements (MGE) | Evidence of presence in low- and middle-income countries (LMICs) recreational environments of ARGs | Carriage of multiple beta-lactamase genes was detected in all isolates except two, including six isolates carrying blaNDM-1. Most detected antibiotic resistance genes (ARGs) were located within a diverse landscape of plasmids, insertion sequences and transposons, including the presence of ISKpn14 upstream of bla_NDM-1_ in a first report in Africa. results confirmed the four beaches are contaminated with bacteria carrying clinically relevant ARGs associated with mobile genetic elements (MGE), which could promote the transmission of ARGs at the recreational water-human interface. | *Evidence(s) of link*  AMR exposure risk in LMIC recreational waters | (Oyelade et al. 2024) |
| Vulnerability (e-e, s, cp) | Ex. | Antibiotic-resistant bacteria  Coastal waters  Surfers  *Escherichia coli*  CTX-M | *Primary data from:*  Recreational water samples and users (surfers) and non-users' gut samples | Proportion and abundance of ARGs, risk of exposure to ARB | Recreational water users (surfers) are at risk of exposure to and colonisation by clinically important antibiotic-resistant E. coli in coastal waters. | From environmental samples, the percentage of bla_CTX-M_-bearing *E. coli* in bathing waters was low (0.07%). It was estimated that in 2015, 2.5M water sports sessions risk ingesting at least resistant bla_CTX-M_-bearing E. coli. In the epidemiological survey, 9/143 (6.3%) surfers were colonised by bla_CTX-M_-bearing *E. coli*, as compared to 2/130 (1.5%) of non-surfers (risk ratio = 4.09, 95% CI: 1.02 -16.4, p = 0.046). | *Evidence(s) link:* potential Health risks from environmental ARB  *Recommendation(s) for action:*  Further work must be done to establish the acquisition of ARB from coastal waters and other natural environments, which have been identified as important reservoirs of ARB. | (Leonard et al. 2018) |
| Vulnerability (e, s, cp) | Ex. | Antimicrobial resistance  Extended-spectrum beta-lactamase  Recreational water  Public health  Carbapenem resistance | *Primary data from:*  Recreational water samples and users (surfers) and non-users' gut samples | Plasmid replicons detected, proportion and abundance of ARB, risk of exposure to ARB | This study demonstrates the occurrence of studied ARB in healthy participants in Ireland. Recreational exposure to bathing water in Ireland was associated with a decreased prevalence of colonisation of the selected ARB. | A total of 80 Enterobacterales were isolated from 73 participants. ESBL-PE were detected in 29 (7.1 %) participants (7 WU, 22 controls), and CRE were detected in nine (2.2 %) participants (4 WU, 5 controls). No carbapenemase-producing Enterobacterales (CPE) were detected. WU were significantly less likely to harbour ESBLPE than controls (risk ratio = 0.34, 95 % CI 0.148 to 0.776, χ2 7.37, p = 0.007). | *Evidence(s) of link:* Heterogeneity in individual study findings on health risks from environmental ARB  *Recommendation(s) for action:*  Further research is needed to elucidate the environmental AMR risks and microbiological mechanisms underpinning gut colonisation by AMR bacteria to understand the risk of AMR transmission in natural environments | (Farrell et al. 2023) |
| Vulnerability (e-e, s, cp) | Ex. | Antibiotic-resistant bacteria  Third-generation cephalosporin (3GCs)  Escherichia coli  Coastal waters  Water sports | *Primary data from:*  Recreational water samples  *Secondary data from*  Observational | Mean number of ARB ingested per swimming session | Estimate human exposure to ARB via water ingested during different water sports. | Despite the low prevalence of resistance to 3GCs amongst *E. coli* in surface waters, there is an identifiable human exposure risk for water users, which varies with the type of water sport undertaken. The relative importance of this exposure is likely to be greater in areas where a large proportion of the population enjoys water sports. In England and Wales, over 6.3 million water sport sessions occurred in 2012 that resulted in the ingestion of at least one 3GCREC. However, this is expected to be a significant underestimate of recreational exposure to all ARB in seawater. | *Evidence(s) of link:* Estimation of exposure to environmental AMR | (Leonard et al. 2015) |
| Vulnerability (e-e, s, cp) | Re. | Antibiotic resistance  Antimicrobial-resistant bacteria  Antimicrobial-resistant genes  Recreational exposures  Aquatic microbial community  Aquatic microbiome  Wastewater  Human health risk  Ambient water  Surface water | *Secondary data from:*  Literature review references | Abundance and presence of ARB, ARGs, infections, colonisation… | The results from this review suggest that exposure to environmental ARB might be a potential source of resistant infections for humans. | Quantitative and qualitative summary of existing evidence of health impacts (colonisation, infection) from exposure in recreational waters by environmental ARB. | *Recommendation(s) for action:*  More data is needed to determine the abundance of antibiotics, active pharmaceutical ingredients, and AMR bacteria and ARGs being discharged into surface waters used for recreation. Surface water monitoring and/or targeted surveillance data on the prevalence, concentration, and location of AMR bacteria are key to beginning to develop human health risk assessments and prioritising the AMR risks to human populations. | (Nappier et al. 2020) |
| Vulnerability (e-e, s, cp)  *Action* | Re. | Antibiotic resistance  Health  Colonisation  Infection  Trasmission  Exposure,  Water  Air  Soil  Food | *Secondary data from.*  Literature references | ARGs, ARB, colonisation, infection… | The study provided two linked systematic maps to synthesise the main research gaps in the context of transmission to humans of environmental AMR. These maps are aimed at policymakers and research funders. | Results from Map 1 showed that consumption/ingestion was the most studied transmission route. Exposure (n = 17), infection (n = 16), colonisation (n = 11) and mortality (n = 2). In addition, *E. coli* was the most highly studied bacterium (n = 16).  For Map 2, the most highly researched species was mixed communities (n = 32). The most common methodology was phenotypic testing (n = 37). The reported outcomes were: characterisation of ARBs (n = 40), ARGs (n = 35). MGEs (n=15) and point mutations (n=1). And the researchers focused on aquatic environments. | *Recommendation(s) for action:*  More structural surveillance of environmental AMR | (Stanton et al. 2022) |
| Vulnerability (e-e, s, cp) | Ob. | Methicillin-resistant Staphylococcus aureus (MRSA)  Antibiotic resistance  Racial disparities  Social determinants of health | *Secondary data from:*  Data from the surveillance system (MRSA cohort) | MRSA identified in blood cultures | This study demonstrates the relationship between racial disparity and rates of invasive community-associated MRSA. This is largely explained by socioeconomic factors (e.g., poverty, overcrowding). | Annual invasive community-associated MRSA incidence was 4.59 per 100,000 among whites and 7.60 per 100,000 among blacks (rate ratio [RR], 1.66; 95% confidence interval [CI], 1.52-1.80). In the mediation analysis, after accounting for census tract-level measures of federally designated medically underserved areas, education, income, housing value, and rural status, 91% of the original racial disparity was explained; no significant association of black race with community-associated MRSA remained (RR, 1.05; 95% CI, .92–1.20) | *Evidence(s) of link:*  Risk of antibiotic resistance infections and the racial disparities | (See et al. 2017) |
| Vulnerability (e-e, s, cp) | Re. | Poverty  Antimicrobial stewardship,  Drug resistance | *Secondary data from.*  Literature references | Colonisation and infection of ARB | Results suggest that addressing social determinants of poverty worldwide remains a crucial yet neglected step towards preventing antimicrobial resistance | The study provides tables summarising the evidence of the following results: Crowding and homelessness were associated with antimicrobial resistance in community and hospital patients. In high-income countries, low income was associated with *Streptococcus pneumoniae* and *Acinetobacter baumannii* resistance and a seven-fold higher infection rate. In low-income countries, the findings on this relation were contradictory. Lack of education was linked to resistant *S. pneumoniae* and *Escherichia coli*. | *Evidence(s) of link:*  Risk of antibiotic-resistant infections and poverty | (Alividza et al. 2018) |
| Vulnerability (e-e, s, cp) | MM | Drug resistance  Microbial  Antibacterial agents  Social conditions  Social determinants of health  Latin America | *Secondary data from.*  Literature references and observational | AMR rate | Socioeconomic factors beyond health care and hospital settings may affect the emergence and dissemination of antimicrobial resistance. | The analysis showed an overall antimicrobial resistance rate of 32.5%, with the highest rates for *S. aureus* (40.6%) and the lowest for *E. coli* (25.7%). We found a small but consistent negative association between socioeconomic factors (income, education, and occupation) and overall antimicrobial resistance in univariate (p < 0.01) and multivariate analyses (p < 0.01), driven by resistant *P. aeruginosa* and *S. aureus*. | *Evidence(s) of link:*  Socioeconomic factors and AMR  *Recommendation(s) for action:*  Preventing and controlling AMR requires efforts above and beyond reducing antibiotic consumption. | (Allel 2021) |
| Vulnerability (e-e, s, cp) | MM | Atimicrobial resistance  Antimicrobial-resistant organisms  Area deprivation index  Social vulnerability index  Sociodemographic factors. | *Secondary data from.*  Literature references and observational | AMR prevalence | Findings revel link between deprivation ARB prevalence in clinical patients | Significant clusters of AMR organisms in areas with high levels of deprivation, as measured by the area deprivation index (ADI). We found a significant spatial autocorrelation between ADI and the prevalence of AMR organisms, particularly for AmpC β-lactamase and methicillin-resistant Staphylococcus aureus, with 14% and 13%, respectively, of the variability in prevalence rates being attributable to their relationship with the ADI values of the neighboring locations | *Evidence(s) of link:*  Socioeconomic factors and AMR | (Cooper et al. 2024) |
| Vulnerability (e-e, s, cp) | Ex. | Bioaerosol  Wastewater treatment plants  Bottom microporous aeration system  Human pathogens  Health risk | *Primary data from:*  Environmental samples (Bioaerosols from WWTP) | Bacterial concentration | AAO (Anaerobic-Anoxic-Oxic) tank posed the highest exposure risk to bacteria in WWTP | The bacterial concentrations are higher in spring than that in winter, with the AAO (Anaerobic-Anoxic-Oxic) tank posing the highest exposure risk during the spring season. The dominant genera in the air samples include *Cutibacterium, Lawsonella*, *Acinetobacter, Pseudomonas*, and Aeromonas. Among the identified genus, 139 bacterial genera were identified as potential human pathogens like *Neisseria, Moraxella, Haemophilus, Escherichia-Shigella* and *Streptococcus.* | *Evidence(s) of link:* Occupational exposure and risk of AMR | (Y. Zhao et al. 2024) |
| Vulnerability (e-e, s, cp) | Ex. | Antibiotic resistance genes; Human health; Microbial community; Nasopharynx; One-Health; Poultry farm | *Primary data from:*  Environmental samples (Bioaerosols from chicken farm) | Bacterial concetration | High concentration of bacteria in aerosols | In total, 116 ARG subtypes and MGEs were identified in the poultry farm. The total bacterial concentration of aerosols inside the chicken house (3.117 × 10^4^ CFU/m^3^) exceeded the corresponding limit. The microbial communities in the samples of cloaca swab (C) and the workers' nasopharyngeal swab (N) were closer, while the abundance distribution of ARGs/ MGEs in cloacal swab (C) and aerosol (AI) in chicken house were much similar. There were certain consistency of the microbial community structure and the distribution of ARGs among the three groups of chicken cloaca, air aerosol, and workers' nasopharynx. | *Evidence(s) of link:* Occupational exposure and risk of AMR | (Yang et al. 2021) |
| Vulnerability (e-e, s, cp) | Ex. | Antibiotic resistance Wastewater treatment plant  Metagenomics Transmission | *Primary data from:*  Water, air, and stool sample, and questionnaires | ARGs, bacterial composition | No increased ARG abundance in WWTP workers. Country of residence and recent antibiotic intake were the strongest contributors to total ARG abundance. | ontrolling for potential confounders, neither the total antibiotic resistance gene (ARG) abundance, nor the overall bacterial composition were significantly different between the two groups. If anything, the ARG richness was slightly lower in WWTP workers, and in a stratified analysis the total ARG abundance was significantly lower in Dutch workers compared to Dutch control participants. | *Evidence(s) of link:*  Conflicting results of occupational exposure and risk of AMR | (Berglund et al. 2023) |
| Vulnerability (e-e, s, cp) | Ex. | Landfill  BIOGAVAL method  AWaRE qualification  Antibiotic-resistant bacteria  Bacteria bioaerosol  Antibiotic resistance | *Primary data from:*  Environmental samples (Bioaerosols from landfill) | Multi-resistant bacteria, antibiotic-resistance in bacterial bioaerosols | The study found antibiotic-resistant bacteria in the bacterial bioaerosol of a landfill and its surroundings. The average concentrations obtained from bacterial bioaerosols, as well as antibiotic-resistant bacteria, were high, which could pose a threat to human health, particularly to landfill workers and communities living in the landfill surroundings. | The study presents a biological Risk assessment by the Biogaval method. Results show that exposure to *S. aureus* (antibiotic resistant) had a more significant impact on the workers' health for respiratory system and skin infections. | *Evidence(s) of link:* Occupational exposure and risk of AMR | (Morgado-Gamero et al. 2021) |
| Vulnerability (e-e, s, cp) | Mo. | NA | *Secondary data from:*  Observational databases (multiple) | Abundance of ARGs in human faecal metagenomes | Increased access to improved water and sanitation was associated with a decrease in ARG | We identified 1589 metagenomes from 26 countries. The mean abundance of ARGs, in units of log10 ARG fragments per kilobase per million mapped reads classified as bacteria, was highest in Africa compared with Europe (p=0·014), North America (p=0·0032), and the Western Pacific (p=0·011), and second highest in South-East Asia compared with Europe (p=0·047) and North America (p=0·014). Increased access to improved water and sanitation was associated with lower ARG abundance (effect estimate –0·22, [95% CI –0·39 to –0·05]) and the association was stronger in urban (–0·32 [–0·63 to 0·00]) than in rural (–0·16 [–0·38 to 0·07]) areas. | *Evidence(s) of link:* Occupational exposure and risk of AMR | (Fuhrmeister et al. 2023) |
| Vulnerability (e-e, s, cp) | Ex. | Antibiotic resistance genes Chlorine-resistant Drinking water system Resistance risk ranking in drinking water system (R3DW)  Risk assessment  Trace level antibiotics | *Primary data from:*  Environmental samples (Bioaerosols from landfill) | ARGs and ARB prevalence | Drinking water reservoirs can act as reservoirs of clinically relevant ARB and ARGs and conventional disinfection methods (e.g., low-level chlorine) may be insufficient to control resistant pathogens | Fourteen ARGs were detected with a relative concentration range of 10⁻⁴–10⁻³ (ARGs/16S rRNA gene). Five isolated ARB were identified as human opportunistic pathogens, one of which (Pseudomonas aeruginosa HLS-6, CCTCC AB 2017269) is resistant to hundreds of milligrams per liter levels of antibiotics and low-level chlorine. This result indicated that ARB tolerant to high-levels of antibiotics could be isolated from environments containing trace levels of antibiotics. Moreover, complete genome sequencing confirmed the inclusion of ARGs (sul1, aadA2) on the class I integron in HLS-6, indicating that the risk of ARGs in this drinking water reservoir could be classified as resistance risk ranking in drinking water system 1 (R3DW 1). | *Evidence(s) of link:*  Drinking water reservoir of ARB and ARGs | (Hu et al. 2021) |
| Vulnerability (e-e, s, cp)  *Action* | Rep. | NA | *Secondary data from:*  Literature references | Infection prevalence | Social determinants of health, such as those related to socioeconomic factors and health disparities, may amplify, moderate, or otherwise influence climate related health effects, particularly when these factors occur simultaneously or close in time or space. | The report provides a comprehensive analysis and discussion of the social factors amplifying the health effects of climate change, identifies vulnerable groups and provides potential areas for action. | *Evidence(s) of link:*  Specific determinants of health and amplified climate effects  *Recommendation(s) for action:*  GIS studies identify vulnerable populations and support targeted interventions and adaptation strategies.  Need to develop valid vulnerability indicators from population data, understand cumulative stress and psychological impacts, and assess resilience measures at individual, institutional, and community levels.. | (Gamble et al. 2016) |
| Vulnerability (e-e, s, cp) | Re. | NA | *Secondary data from:*  Literature references | NA | The study found that wildfires pose a significant hazard to respiratory health, especially for individuals with asthma. | The review offers qualitative and quantitative evidence of associations between wildfires and upper and lower respiratory effects, including infections as well as exacerbations of asthma and chronic obstructive pulmonary disease. | *Evidence(s) of link:*  Wildfires and respiratory diseases | (Weheba et al. 2024) |
| Vulnerability (e-e, s, cp) | Ex. | NA | *Primary data from:*  Samples from mussels and oysters’ tissue and liquor | Occurrence of ARB and ARGs | Bacterial contamination was most often detected in bivalves collected near point sources of human effluent, and AMR *E. coli* was only detected in samples from this area. | For *E. coli*-positive sites, the proportion of surrounding agricultural land was slightly lower (0.42 vs. 0.47) and the distance to human effluent was shorter (3085.8 vs. 3553.3). For AMR-positive isolates, the difference was more pronounced: less agricultural land (0.39 vs. 0.42) and much closer proximity to human effluent (1247.8 vs. 4286.6). | *Evidence(s) of link:*  AMR in oysters/mussels and human effluent | (Rees et al. 2015) |
| Vulnerability (e-e, s, cp) | MM | Blue mussels  Oysters  Scallops  Faecal indicator  Bivalve production  Food safety  ST131 | *Primary data from:*  Samples from mussels and oysters’ tissue and liquor | Prevalence of AMR, occurrence of ARB | Potential risk for transmission of resistant and pathogenic *E. coli* to the consumers of raw seafood. | Among 261 E. coli-positive samples, 8.4% of isolates showed resistance to at least one antimicrobial, most commonly tetracycline (5.7%), ampicillin (4.6%), and sulfamethoxazole (3.1%). Selective screening identified ESC-resistant E. coli in 3.3% of samples, QREC in 12.8%, and no CRE. The bla_CTX-M-15_ gene was the most prevalent mechanism of ESC resistance, occasionally co-occurring with blaCMY-42, with additional contributions from bla_CTX-M-3_, blaCTX-M-1, and an AmpC mutation. QREC exhibited significant spatial clustering, and samples exceeding the Class A E. coli threshold were associated with higher odds of detecting ESC-resistant E. coli or QREC, though several resistant isolates were also found below this threshold. Whole-genome sequencing revealed that some ESC-resistant isolates originated from seafood commonly consumed raw, including one high-risk ST131 clone carrying the plasmid-encoded senB gene linked to diarrhoeal disease. | *Evidence(s) of link:*  Consumption of raw seafood and risk of AMR | (Svanevik et al. 2023) |
| Vulnerability (e-e, s, cp) | Ex. | AMR  Fresh produce  ARGs  ARB  Plasmidome | *Primary data from:*  Samples from vegetables | Occurrence of ARB and ARGs | Presence of ARGs in almost all fresh vegetable samples | In total, 91 ARB were isolated from fresh produce, mainly cephalosporin-resistant Enterobacterales (n = 64) and carbapenem-resistant *P. aeruginosa* (n = 13). All P. aeruginosa, as well as 16 Enterobacterales' isolates were multidrug-resistant. No differences between imported and Swiss fresh produce were found regarding the number of ARB. In 95 % of samples at least one ARG was detected, being the most frequent sul1, bla_TEM_, and ermB. | *Evidence(s) of link:*  Consumption of raw vegetables and risk of AMR | (Kläui et al. 2024) |
| Vulnerability (e-e, s, cp) | Mo. | NA |  | Deaths associated with AMR and attributable death to AMR | Trends in AMR mortality over the past 31 years varied substantially by age and location. | From 1990 to 2021, deaths from AMR decreased by more than 50% among children younger than 5 years yet increased by over 80% for adults 70 years and older. AMR mortality decreased for children younger than 5 years in all super-regions, whereas AMR mortality in people 5 years and older increased in all super-regions.  Super-regions with the highest all-age AMR mortality rate in 2050 are forecasted to be south Asia and Latin America and the Caribbean. Increases in deaths attributable to AMR will be largest among those 70 years and older (65·9% [61·2–69·8] of all-age deaths attributable to AMR in 2050).. | *Evidence(s) of link:*  AMR mortality and age and location. | (Naghavi et al. 2024) |
| Vulnerability (e-e, s, cp) | Re. | NA | *Secondary data from:*  Literature refererences | ARB and ARGs | The review highlights the risk of AMR related infections in older adults and identified LTCF as AMR reservoirs. | The review explored factors contributing to the elevated risk of AMR-related infections in the elderly, ranging from age-related immunological decline to higher antibiotic consumption driven by increased susceptibility to specific infections, such as urinary tract infections. The study also highlighted long-term care facilities (LTCF) as potential reservoirs of AMR for this population group. | *Evidence(s) of link:*  *AMR-related infections and older adults* | (Augustine and Bonomo 2011) |
| Vulnerability (e-e, s, cp) | Re. | NA | *Secondary data from:*  Literature references | Mixed (ARB, ARGS, infections prevalence, non-communicable disease prevalence) |  | The review provided a summary table for the mechanisms of the most prevalent (multidrug-resistant organisms) MDROs and their most common infections in the elderly. Highlights how aging-related factors like immunosenescence, frailty, and multimorbidity increase the burden of infections from MDROs in this population | *Evidence(s) of link:*  Aging-related factors and *AMR* | (Theodorakis et al. 2024) |
| Vulnerability (e-e, s, cp) | Re. | Asthma  Iinfection  Inflammation  Autoimmunity  Disease  Multimorbidity  Comorbidity  Risk  Epidemiology | *Secondary data from:*  Literature references | Prevalence of infections | The study found asthma is associated with the increased risk of a broad range of respiratory infections | The study found asthma is associated with the increased risk of a broad range of respiratory infections including pneumococcal pneumonia, IPD, Streptococcus pyogenesupper respiratory infection (URI), recurrent or persistent otitis media, acute otitis media/myringitis, pertussis, viral URI, pneumonia, sinusitis, H1N1 influenza, and breakthrough varicella infection. These respiratory infections represent clinically both common and serious infections, microbiologically both bacterial (gram positive and gram negative) and viral infections, and anatomically both upper and lower airway infection/pneumonia. | *Evidence(s) of link:*  Asthma and respiratory tract infections | (Kwon et al. 2021) |
| Vulnerability (e-e, s, cp) | Ob. | Diabetes mellitus  Hospitalization  Infection | *Secondary data from:*  Observational data (Cohort study) | Infection prevalence | Population with diabetes at higher risk of infections | Diabetes group had a greater risk of almost all the types of infections considered, with the adjusted IRRs (aIRRs) for infection-related hospitalizations being the highest for hepatic abscess (aIRR, 10.17; 95% confidence interval [CI], 7.04 to 14.67), central nervous system (CNS) infections (aIRR, 8.72; 95% CI, 6.64 to 11.45), and skin and soft tissue infections other than cellulitis (SSTIs) (aIRR, 3.52; 95% CI, 3.20 to 3.88). Diabetes group also had a greater risk of ICU admission and death due to SSTIs (aIRR, 11.75; 95% CI, 7.32 to 18.86), CNS infections (aIRR, 5.25; 95% CI, 3.53 to 7.79), and bone and joint infections (aIRR, 4.78; 95% CI, 3.09 to 7.39). | *Evidence(s) of link:*  Diabetes and *risk of infections* | (Kim et al. 2019) |
| Vulnerability (e-e, s, cp) | Re. | Diabetes mellitus  Hospitalization  Infection | *Secondary data from:*  Literature references | Infection prevalence | People with type 2 diabetes mellitus (T2DM) were twofold more likely to have infections including resistant infections | People with type 2 diabetes mellitus (T2DM) were twofold more likely to have urinary tract (OR=2.42; 95% CI 1.83 to 3.20; I2 19.1%) or respiratory (OR=2.35; 95% CI 1.49 to 3.69; I2 58.1%) resistant infections | *Evidence(s) of link:*  Diabetes and *risk of AMR related infections* | (Carrillo-Larco et al. 2022) |
| Vulnerability (e-e, s, cp) | Re. | Intimicrobial resistance  Implementation science  Barriers and facilitators  Rational antibiotic use | *Secondary data from:*  Literature references | NA | Identification of main intervention areas in LMICs | The review highlights the main intervention areas in LMICs including addressing constraints in resources and the infrastructure of the facilities where interventions were implemented; lack of national initiatives and policies on antibiotic use and reluctance to change prescribing behaviours. | *Evidence(s) of link:*  *Recommendation(s) for action:* | (Wu et al. 2022) |
| *Action* | Ex. | NA | *Primary data from:*  Data from interviews and focus group discussions | NA | The study found successes and challenges of building resilience in community-based health systems in Ethiopia during the drought. | The study identifies key themes: 1) organised community groups linked to the health system, 2) an effective community health workforce within strong health systems, 3) adaptable human resource structures and service delivery models, 4) training and preparedness, and 5) strong government leadership with decentralized decision making | *Recommendation(s) for action:*  Community integration and workforce capacity. Governance and system resilience. | (Rawat et al. 2022) |

# **References**

Ahmed, Warish, Pradip Gyawali, Kerry A. Hamilton, et al. 2021. ‘Antibiotic Resistance and Sewage-Associated Marker Genes in Untreated Sewage and a River Characterized During Baseflow and Stormflow’. *Frontiers in Microbiology* 12. https://www.frontiersin.org/article/10.3389/fmicb.2021.632850.

Ahmed, Warish, Qian Zhang, Aldo Lobos, et al. 2018. ‘Precipitation Influences Pathogenic Bacteria and Antibiotic Resistance Gene Abundance in Storm Drain Outfalls in Coastal Sub-Tropical Waters’. *Environment International* 116 (July): 308–18. https://doi.org/10.1016/j.envint.2018.04.005.

Al Aukidy, M., and P. Verlicchi. 2017. ‘Contributions of Combined Sewer Overflows and Treated Effluents to the Bacterial Load Released into a Coastal Area’. *Science of The Total Environment* 607–608 (December): 483–96. https://doi.org/10.1016/j.scitotenv.2017.07.050.

Alividza, Vivian, Victor Mariano, Raheelah Ahmad, et al. 2018. ‘Investigating the Impact of Poverty on Colonization and Infection with Drug-Resistant Organisms in Humans: A Systematic Review’. *Infectious Diseases of Poverty* 7 (1): 76. https://doi.org/10.1186/s40249-018-0459-7.

Allel, Kasim. 2021. ‘Exploring the Relationship between Climate Change and Antimicrobial-Resistant Bacteria: To What Extent Does This Present a Current and Long-Term Threat to Population Health?’ *The International Journal of Climate Change: Impacts and Responses*, ahead of print, January 1. https://doi.org/10.18848/1835-7156/CGP/v13i01/27-37.

Anikeeva, Olga, Alana Hansen, Blesson Varghese, et al. 2024. ‘The Impact of Increasing Temperatures Due to Climate Change on Infectious Diseases’. Clinical Review. *BMJ* 387 (October): e079343. https://doi.org/10.1136/bmj-2024-079343.

Augustine, S., and R. A. Bonomo. 2011. ‘Taking Stock of Infections and Antibiotic Resistance in the Elderly and Long-Term Care Facilities: A Survey of Existingand Upcoming Challenges’. *European Journal of Microbiology and Immunology* 1 (3): 190–97. https://doi.org/10.1556/EuJMI.1.2011.3.2.

Berglund, Fanny, Daloha Rodríguez-Molina, Gratiela Gradisteanu Pircalabioru, et al. 2023. ‘The Resistome and Microbiome of Wastewater Treatment Plant Workers – The AWARE Study’. *Environment International* 180 (October): 108242. https://doi.org/10.1016/j.envint.2023.108242.

Bollinger, E., J.P. Zubrod, F.Y. Lai, et al. 2021. ‘Antibiotics as a Silent Driver of Climate Change? A Case Study Investigating Methane Production in Freshwater Sediments’. *Ecotoxicology and Environmental Safety* 228 (December): 113025. https://doi.org/10.1016/j.ecoenv.2021.113025.

Carney, Richard L., Maurizio Labbate, Nachshon Siboni, Kaitlin A. Tagg, Simon M. Mitrovic, and Justin R. Seymour. 2019. ‘Urban Beaches Are Environmental Hotspots for Antibiotic Resistance Following Rainfall’. *Water Research* 167 (December): 115081. https://doi.org/10.1016/j.watres.2019.115081.

Carrillo-Larco, Rodrigo M, Cecilia Anza-Ramírez, Giancarlo Saal-Zapata, et al. 2022. ‘Type 2 Diabetes Mellitus and Antibiotic-Resistant Infections: A Systematic Review and Meta-Analysis’. *Journal of Epidemiology and Community Health* 76 (1): 75–84. https://doi.org/10.1136/jech-2020-216029.

Chadsuthi, Sudarat, Karine Chalvet-Monfray, Anuwat Wiratsudakul, and Charin Modchang. 2021. ‘The Effects of Flooding and Weather Conditions on Leptospirosis Transmission in Thailand’. *Scientific Reports* 11 (1): 1486. https://doi.org/10.1038/s41598-020-79546-x.

Chase, Jonathan M., and Tiffany M. Knight. 2003. ‘Drought-Induced Mosquito Outbreaks in Wetlands’. *Ecology Letters* 6 (11): 1017–24. https://doi.org/10.1046/j.1461-0248.2003.00533.x.

Cooper, Lauren N., Alaina M. Beauchamp, Tanvi A. Ingle, et al. 2024. ‘Socioeconomic Disparities and the Prevalence of Antimicrobial Resistance’. *Clinical Infectious Diseases: An Official Publication of the Infectious Diseases Society of America* 79 (6): 1346–53. https://doi.org/10.1093/cid/ciae313.

Downs, Joni, Jim Downs, Victor Mesev, and Saurav Chakraborty. 2025. ‘Climate-Induced Expansion of Lyme Disease in East Central Ohio’. *International Journal of Environmental Health Research*, January 29, 1–11. https://doi.org/10.1080/09603123.2025.2456966.

Drozdova, Jarmila, Helena Raclavska, and Hana Skrobankova. 2015. ‘A Survey of Heavy Metals in Municipal Wastewater in Combined Sewer Systems during Wet and Dry Weather Periods’. *Urban Water Journal*, February 17. world. https://www.tandfonline.com/doi/abs/10.1080/1573062X.2013.831913.

Eber, Michael R., Michelle Shardell, Marin L. Schweizer, Ramanan Laxminarayan, and Eli N. Perencevich. 2011. ‘Seasonal and Temperature-Associated Increases in Gram-Negative Bacterial Bloodstream Infections among Hospitalized Patients’. *PLoS ONE* 6 (9): e25298. https://doi.org/10.1371/journal.pone.0025298.

Farrell, Maeve Louise, Alexandra Chueiri, Louise O’Connor, et al. 2023. ‘Assessing the Impact of Recreational Water Use on Carriage of Antimicrobial Resistant Organisms’. *Science of The Total Environment* 888 (August): 164201. https://doi.org/10.1016/j.scitotenv.2023.164201.

Fisman, David, Eleni Patrozou, Yehuda Carmeli, et al. 2014. ‘Geographical Variability in the Likelihood of Bloodstream Infections Due to Gram-Negative Bacteria: Correlation with Proximity to the Equator and Health Care Expenditure’. *PLOS ONE* 9 (12): e114548. https://doi.org/10.1371/journal.pone.0114548.

Fuhrmeister, Erica R., Abigail P. Harvey, Maya L. Nadimpalli, et al. 2023. ‘Evaluating the Relationship between Community Water and Sanitation Access and the Global Burden of Antibiotic Resistance: An Ecological Study’. *The Lancet Microbe* 4 (8): e591–600. https://doi.org/10.1016/S2666-5247(23)00137-4.

Furlan, João Pedro Rueda, Fábio Parra Sellera, Nilton Lincopan, Daniela Debone, Simone Georges El Khouri Miraglia, and Ronan Adler Tavella. 2024. ‘Catastrophic Floods and Antimicrobial Resistance: Interconnected Threats with Wide-Ranging Impacts’. *One Health* 19 (December): 100891. https://doi.org/10.1016/j.onehlt.2024.100891.

Gamble, J. L., J. Balbus, M. Berger, et al. 2016. ‘Ch. 9: Populations of Concern’. In *The Impacts of Climate Change on Human Health in the United States: A Scientific Assessment*. U.S. Global Change Research Program, Washington, DC. https://health2016.globalchange.gov/node/16/index.html.

Hammer, Tobin J., Noah Fierer, Bess Hardwick, et al. 2016. ‘Treating Cattle with Antibiotics Affects Greenhouse Gas Emissions, and Microbiota in Dung and Dung Beetles’. *Proceedings of the Royal Society B: Biological Sciences* 283 (1831): 20160150. https://doi.org/10.1098/rspb.2016.0150.

Hashimoto, Mayuko, Haruka Hasegawa, and Sumio Maeda. 2019. ‘High Temperatures Promote Cell-to-Cell Plasmid Transformation in Escherichia Coli’. *Biochemical and Biophysical Research Communications* 515 (1): 196–200. https://doi.org/10.1016/j.bbrc.2019.05.134.

Hassan, Brekhna, Muhammad Ijaz, Asadullah Khan, et al. 2021. ‘A Role for Arthropods as Vectors of Multidrug-Resistant Enterobacterales in Surgical Site Infections from South Asia’. *Nature Microbiology* 6 (10): 1259–70. https://doi.org/10.1038/s41564-021-00965-1.

Hellberg, Rosalee S., and Eric Chu. 2016. ‘Effects of Climate Change on the Persistence and Dispersal of Foodborne Bacterial Pathogens in the Outdoor Environment: A Review’. *Critical Reviews in Microbiology* 42 (4): 548–72. https://doi.org/10.3109/1040841X.2014.972335.

Hu, Yaru, Lei Jiang, Xiaoyan Sun, et al. 2021. ‘Risk Assessment of Antibiotic Resistance Genes in the Drinking Water System’. *Science of The Total Environment* 800 (December): 149650. https://doi.org/10.1016/j.scitotenv.2021.149650.

Jang, Jiyi, Minjeong Kim, Sangsoo Baek, et al. 2021. ‘Hydrometeorological Influence on Antibiotic‐Resistance Genes (ARGs) and Bacterial Community at a Recreational Beach in Korea’. *Journal of Hazardous Materials* 403 (February): 123599. https://doi.org/10.1016/j.jhazmat.2020.123599.

Kaba, Hani E. J., Ellen Kuhlmann, and Simone Scheithauer. 2020. ‘Thinking Outside the Box: Association of Antimicrobial Resistance with Climate Warming in Europe – A 30 Country Observational Study’. *International Journal of Hygiene and Environmental Health* 223 (1): 151–58. https://doi.org/10.1016/j.ijheh.2019.09.008.

Kim, Eun Jin, Kyoung Hwa Ha, Dae Jung Kim, and Young Hwa Choi. 2019. ‘Diabetes and the Risk of Infection: A National Cohort Study’. *Diabetes & Metabolism Journal* 43 (6): 804–14. https://doi.org/10.4093/dmj.2019.0071.

Kläui, Anita, Ueli Bütikofer, Javorka Naskova, Elvira Wagner, and Elisabet Marti. 2024. ‘Fresh Produce as a Reservoir of Antimicrobial Resistance Genes: A Case Study of Switzerland’. *Science of The Total Environment* 907 (January): 167671. https://doi.org/10.1016/j.scitotenv.2023.167671.

Kwon, Jung Hyun, Chung-Il Wi, Hee Yun Seol, et al. 2021. ‘Risk, Mechanisms and Implications of Asthma-Associated Infectious and Inflammatory Multimorbidities (AIMs) among Individuals With Asthma: A Systematic Review and a Case Study’. *Allergy, Asthma & Immunology Research* 13 (5): 697–718. https://doi.org/10.4168/aair.2021.13.5.697.

Leonard, Anne F. C., Lihong Zhang, Andrew J. Balfour, et al. 2018. ‘Exposure to and Colonisation by Antibiotic-Resistant *E. Coli* in UK Coastal Water Users: Environmental Surveillance, Exposure Assessment, and Epidemiological Study (Beach Bum Survey)’. *Environment International* 114 (May): 326–33. https://doi.org/10.1016/j.envint.2017.11.003.

Leonard, Anne F. C., Lihong Zhang, Andrew J. Balfour, Ruth Garside, and William H. Gaze. 2015. ‘Human Recreational Exposure to Antibiotic Resistant Bacteria in Coastal Bathing Waters’. *Environment International* 82 (September): 92–100. https://doi.org/10.1016/j.envint.2015.02.013.

Li, Weibin, Chaojie Liu, Hung Chak Ho, et al. 2023. ‘Association between Antibiotic Resistance and Increasing Ambient Temperature in China: An Ecological Study with Nationwide Panel Data’. *The Lancet Regional Health. Western Pacific* 30 (January): 100628. https://doi.org/10.1016/j.lanwpc.2022.100628.

Liu, Zhidong, Guoyong Ding, Ying Zhang, Xin Xu, Qiyong Liu, and Baofa Jiang. 2015. ‘Analysis of Risk and Burden of Dysentery Associated with Floods from 2004 to 2010 in Nanning, China’. *The American Journal of Tropical Medicine and Hygiene* 93 (5): 925–30. https://doi.org/10.4269/ajtmh.14-0825.

MacFadden, Derek R., Sarah F. McGough, David Fisman, Mauricio Santillana, and John S. Brownstein. 2018. ‘Antibiotic Resistance Increases with Local Temperature’. *Nature Climate Change* 8 (6): 510–14. https://doi.org/10.1038/s41558-018-0161-6.

McGough, Sarah F, Derek R MacFadden, Mohammad W Hattab, Kåre Mølbak, and Mauricio Santillana. 2020. ‘Rates of Increase of Antibiotic Resistance and Ambient Temperature in Europe: A Cross-National Analysis of 28 Countries between 2000 and 2016’. *Eurosurveillance* 25 (45): 1900414. https://doi.org/10.2807/1560-7917.ES.2020.25.45.1900414.

Morgado-Gamero, Wendy B., Alexander Parody, Jhorma Medina, Laura A. Rodriguez-Villamizar, and Dayana Agudelo-Castañeda. 2021. ‘Multi-Antibiotic Resistant Bacteria in Landfill Bioaerosols: Environmental Conditions and Biological Risk Assessment’. *Environmental Pollution* 290 (December): 118037. https://doi.org/10.1016/j.envpol.2021.118037.

Naghavi, Mohsen, Stein Emil Vollset, Kevin S Ikuta, et al. 2024. ‘Global Burden of Bacterial Antimicrobial Resistance 1990–2021: A Systematic Analysis with Forecasts to 2050’. *The Lancet* 404 (10459): 1199–226. https://doi.org/10.1016/S0140-6736(24)01867-1.

Nappier, Sharon P., Krista Liguori, Audrey M. Ichida, Jill R. Stewart, and Kaedra R. Jones. 2020. ‘Antibiotic Resistance in Recreational Waters: State of the Science’. *International Journal of Environmental Research and Public Health* 17 (21): 8034. https://doi.org/10.3390/ijerph17218034.

Oyelade, Abolade A., Odion O. Ikhimiukor, Blessing I. Nwadike, Obasola E. Fagade, and Olawale O. Adelowo. 2024. ‘Assessing the Risk of Exposure to Antimicrobial Resistance at Public Beaches: Genome-Based Insights into the Resistomes, Mobilomes and Virulomes of Beta-Lactams Resistant *Enterobacteriaceae* from Recreational Beaches in Lagos, Nigeria’. *International Journal of Hygiene and Environmental Health* 258 (May): 114347. https://doi.org/10.1016/j.ijheh.2024.114347.

Pai, Hsiu-Hua, Wei-Chen Chen, and Chien-Fang Peng. 2005. ‘Isolation of Bacteria with Antibiotic Resistance from Household Cockroaches (Periplaneta Americana and Blattella Germanica)’. *Acta Tropica* 93 (3): 259–65. https://doi.org/10.1016/j.actatropica.2004.11.006.

Pärnänen, Katariina M. M., Carlos Narciso-da-Rocha, David Kneis, et al. 2019. ‘Antibiotic Resistance in European Wastewater Treatment Plants Mirrors the Pattern of Clinical Antibiotic Resistance Prevalence’. *Science Advances* 5 (3): eaau9124. https://doi.org/10.1126/sciadv.aau9124.

Pham, Dung Ngoc, Lerone Clark, and Mengyan Li. 2021. ‘Microplastics as Hubs Enriching Antibiotic-Resistant Bacteria and Pathogens in Municipal Activated Sludge’. *Journal of Hazardous Materials Letters* 2 (November): 100014. https://doi.org/10.1016/j.hazl.2021.100014.

Rawat, Angeli, Jonas Karlstrom, Agazi Ameha, et al. 2022. ‘The Contribution of Community Health Systems to Resilience: Case Study of the Response to the Drought in Ethiopia’. *Journal of Global Health* 12 (October). https://doi.org/10.7189/jogh.12.14001.

Rees, Erin E., Jeff Davidson, John Morris Fairbrother, Sophie St. Hilaire, Matthew Saab, and J T. McClure. 2015. ‘Occurrence and Antimicrobial Resistance of *Escherichia Coli* in Oysters and Mussels from Atlantic Canada’. *Foodborne Pathogens and Disease* 12 (2): 164–69. https://doi.org/10.1089/fpd.2014.1840.

Robas, Marina, Agustín Probanza, Daniel González, and Pedro A. Jiménez. 2021. ‘Mercury and Antibiotic Resistance Co-Selection in Bacillus Sp. Isolates from the Almadén Mining District’. *International Journal of Environmental Research and Public Health* 18 (16): 8304. https://doi.org/10.3390/ijerph18168304.

Rodríguez-Verdugo, Alejandra, Brandon S. Gaut, and Olivier Tenaillon. 2013. ‘Evolution of Escherichia Coli Rifampicin Resistance in an Antibiotic-Free Environment during Thermal Stress’. *BMC Evolutionary Biology* 13 (February): 50. https://doi.org/10.1186/1471-2148-13-50.

Rodríguez-Verdugo, Alejandra, Natalie Lozano-Huntelman, Mauricio Cruz-Loya, Van Savage, and Pamela Yeh. 2020. ‘Compounding Effects of Climate Warming and Antibiotic Resistance’. *iScience* 23 (4): 101024. https://doi.org/10.1016/j.isci.2020.101024.

See, Isaac, Paul Wesson, Nicole Gualandi, et al. 2017. ‘Socioeconomic Factors Explain Racial Disparities in Invasive Community-Associated Methicillin-Resistant Staphylococcus Aureus Disease Rates’. *Clinical Infectious Diseases* 64 (5): 597–604. https://doi.org/10.1093/cid/ciw808.

Stange, C., and A. Tiehm. 2020. ‘Occurrence of Antibiotic Resistance Genes and Microbial Source Tracking Markers in the Water of a Karst Spring in Germany’. *Science of The Total Environment* 742 (November): 140529. https://doi.org/10.1016/j.scitotenv.2020.140529.

Stanton, Isobel Catherine, Alison Bethel, Anne Frances Clare Leonard, William Hugo Gaze, and Ruth Garside. 2022. ‘Existing Evidence on Antibiotic Resistance Exposure and Transmission to Humans from the Environment: A Systematic Map’. *Environmental Evidence* 11 (1): 8. https://doi.org/10.1186/s13750-022-00262-2.

Svanevik, Cecilie Smith, Madelaine Norström, Bjørn Tore Lunestad, Jannice Schau Slettemeås, and Anne Margrete Urdahl. 2023. ‘From Tide to Table: A Whole-Year, Coastal-Wide Surveillance of Antimicrobial Resistance in *Escherichia Coli* from Marine Bivalves’. *International Journal of Food Microbiology* 407 (December): 110422. https://doi.org/10.1016/j.ijfoodmicro.2023.110422.

Theodorakis, Nikolaos, Georgios Feretzakis, Christos Hitas, et al. 2024. ‘Antibiotic Resistance in the Elderly: Mechanisms, Risk Factors, and Solutions’. *Microorganisms* 12 (10): 1978. https://doi.org/10.3390/microorganisms12101978.

Tipper, Holly J., Isobel C. Stanton, Rachel A. Payne, Daniel S. Read, and Andrew C. Singer. 2024. ‘Do Storm Overflows Influence AMR in the Environment and Is This Relevant to Human Health? A UK Perspective on a Global Issue’. *Water Research* 260 (August): 121952. https://doi.org/10.1016/j.watres.2024.121952.

Van Eldijk, Timo J B, Eleanor A Sheridan, Guillaume Martin, Franz J Weissing, Oscar P Kuipers, and G Sander Van Doorn. 2024. ‘Temperature Dependence of the Mutation Rate towards Antibiotic Resistance’. *JAC-Antimicrobial Resistance* 6 (3): dlae085. https://doi.org/10.1093/jacamr/dlae085.

Weheba, Ahmed, Anne Vertigan, Abeer Abdelsayad, and Susan M. Tarlo. 2024. ‘Respiratory Diseases Associated With Wildfire Exposure in Outdoor Workers’. *The Journal of Allergy and Clinical Immunology: In Practice* 12 (8): 1989–96. https://doi.org/10.1016/j.jaip.2024.03.033.

Wei, Nana, Jinmiao Lu, Yi Dong, and Shibo Li. 2022. ‘Profiles of Microbial Community and Antibiotic Resistome in Wild Tick Species’. *mSystems*, ahead of print, August 1. 1752 N St., N.W., Washington, DC. https://doi.org/10.1128/msystems.00037-22.

Westrich, Jason R., Alina M. Ebling, William M. Landing, et al. 2016. ‘Saharan Dust Nutrients Promote Vibrio Bloom Formation in Marine Surface Waters’. *Proceedings of the National Academy of Sciences* 113 (21): 5964–69. https://doi.org/10.1073/pnas.1518080113.

Wu, Shishi, Elias Tannous, Victoria Haldane, Moriah E. Ellen, and Xiaolin Wei. 2022. ‘Barriers and Facilitators of Implementing Interventions to Improve Appropriate Antibiotic Use in Low- and Middle-Income Countries: A Systematic Review Based on the Consolidated Framework for Implementation Research’. *Implementation Science : IS* 17 (May): 30. https://doi.org/10.1186/s13012-022-01209-4.

Yang, Fan, Yanling Gao, Hongcheng Zhao, et al. 2021. ‘Revealing the Distribution Characteristics of Antibiotic Resistance Genes and Bacterial Communities in Animal-Aerosol-Human in a Chicken Farm: From One-Health Perspective’. *Ecotoxicology and Environmental Safety* 224 (November): 112687. https://doi.org/10.1016/j.ecoenv.2021.112687.

Yang, Ji Woo, Ji-Hyun Nam, Kwang Jun Lee, and Jung Sik Yoo. 2024. ‘Effect of Temperature on Carbapenemase-Encoding Plasmid Transfer in Klebsiella Pneumoniae’. *Microorganisms* 12 (3): 454. https://doi.org/10.3390/microorganisms12030454.

Yang, Qiu E., Zhenyan Lin, Dehao Gan, et al. 2025. ‘Microplastics Mediates the Spread of Antimicrobial Resistance Plasmids via Modulating Conjugal Gene Expression’. *Environment International* 195 (January): 109261. https://doi.org/10.1016/j.envint.2025.109261.

Zhang, Rong-min, Xin-lei Lian, Li-wei Shi, et al. 2023. ‘Dynamic Human Exposure to Airborne Bacteria-Associated Antibiotic Resistomes Revealed by Longitudinal Personal Monitoring Data’. *Science of The Total Environment* 904 (December): 166799. https://doi.org/10.1016/j.scitotenv.2023.166799.

Zhang, Shuai, Yue Wang, Hailiang Song, Ji Lu, Zhiguo Yuan, and Jianhua Guo. 2019. ‘Copper Nanoparticles and Copper Ions Promote Horizontal Transfer of Plasmid-Mediated Multi-Antibiotic Resistance Genes across Bacterial Genera’. *Environment International* 129 (August): 478–87. https://doi.org/10.1016/j.envint.2019.05.054.

Zhao, Wenya, Shikan Zheng, Chengsong Ye, Jianguo Li, and Xin Yu. 2024. ‘Nonlinear Impacts of Temperature on Antibiotic Resistance in Escherichia Coli’. *Environmental Science and Ecotechnology* 22 (November): 100475. https://doi.org/10.1016/j.ese.2024.100475.

Zhao, Yulei, Mingyu Xiong, Kinfai Ho, et al. 2024. ‘Bioaerosol Emission and Exposure Risk from a Wastewater Treatment Plant in Winter and Spring’. *Ecotoxicology and Environmental Safety* 287 (November): 117294. https://doi.org/10.1016/j.ecoenv.2024.117294.
